# Knee synovial fluid flow and heat transfer, a power law model

**DOI:** 10.1038/s41598-023-44482-z

**Published:** 2023-10-24

**Authors:** Shahid Hasnain, Imran Abbas, Nawal Odah Al-Atawi, Muhammad Saqib, Muhammad F. Afzaal, Daoud S. Mashat

**Affiliations:** 1 Department of Mathematics, University of Chakwal, Chakwal, Pakistan; 2 Department of Mathematics, Air University, Islamabad, Pakistan; 3 Department of Mathematics, King Abdulaziz University, Jeddah, Postal Code 21589 Saudi Arabia; 4 Department of Mathematics, Tabuk University, Tabuk, Postal Code 71491 Saudi Arabia; 5 Department of Mathematics, Khwaja Fareed University of Engineering and Information Technology (KFUEIT), Rahim Yar Khan, Pakistan; 6 King Abdullah University of Science and Technology, Jeddah, Saudi Arabia

**Keywords:** Biophysics, Mathematics and computing, Physics

## Abstract

For the purpose of understanding, the governing system of partial differential equations for synovial fluid flow velocity and temperature distribution in the knee joint has been successfully solved for the first time. Therefore, such an article is shedding light on the convective diffusion of the viscous flow along the articular surfaces of the joints through the introduction of power-law fluids with different features of permeability, and stagnation point flow along a magnetic field. Henceforth, the frictional energy causes the knee joint’s temperature to increase. By way of filtration, heated synovial fluid reaches the articular cartilage and provides heat to the bone and cartilage. The lubricant in the joint cavity is properly mixed with this cooled fluid. A rectangular region flow and diffusion model is used to define the issue, thermal diffusion and flow inside the intra-articular gap, as well as flow and thermal diffusion within the porous matrix covering the approaching bones at the joint. Using the similarity solution approach, the linked mixed boundary value problem is addressed. The fluid has been shown to resist moving into or out of the cartilage in certain sick and/or aging synovial joints, causing the temperature to increase. By changing the values of the parameters from their usual levels, it is observed that the temperature did increase in aged and sick joints which impact cartilage and/or synovial fluid degradation.

## Introduction

The flow properties of synovial fluid are crucial to understanding the many kinds of lubrication that occur in human knee joints (synovial). In the context of lubrication, visco-elastic and viscous flow properties are most often the ones that are investigated as part of the research process. At a constant shear rate (steady shear flow), such as Couette or pipe Poiseuille flows, $\eta$ of a fluid is a crucial parameter that significantly impacts the behavior of a fluid. The rheological behavior of the fluid is responsible for its deformation under shear stress and is of utmost importance in the calculation of the necessary gap to sustain a specific load in such systems^1^. Plane Couette flow is the steady flow between two infinite parallel plates moving in opposite directions at a constant speed^1^ and pipe Poiseuille flow is when a constant pressure gradient in the axial direction drives a steady flow in an infinitely long circular pipe. The Navier-Stokes equations are a set of equations that describe both flows. These flows are easy to understand which are examples of shear flows and the steady analytical solution is known in both cases^2^. Extensional viscosity and viscoelastic properties help preserve fluid film conditions in these circumstances. Oscillatory movements with a small amplitude as those seen in running and leaping, may benefit greatly from the presence of viscoelasticity^1,2,3^.

Among the several rheological properties, the constant shear viscosity is the most basic one to measure which simulates the aforementioned flows. Note that not all fluids have a constant zero-shear viscosity like Newtonian fluids. Non-Newtonian fluids have shear-thinning or shear-thickening viscosity with shear rate. If the zero-shear viscosity is not constant, more complicated rheological models are needed to describe their behavior. The constant $\eta$ describes the resistance to the flow of simple Newtonian fluids and can be used to describe their behavior in various conditions. In non-Newtonian fluids, the connection between shear stress and rate may not be linear, thus the constant $\eta$ notion may not apply. Such forecasts require careful study of the fluid’s rheology^1^. In fluids with higher complexity, such as synovial fluid, the viscosity does indeed exhibit variations in response to changes in shear rate (${\dot{\gamma }}$). The behavior of synovial fluid is influenced by its distinctive composition and the fundamental principles of fluid dynamics^1^.

The viscosity of the synovial fluid is primarily determined by the random molecular motion within the fluid at low shear rates. In this particular state, the molecules present in the fluid, including larger ones such as hyaluronic acid (HA), demonstrate a propensity to undergo mechanical interlocking or entanglement. The phenomenon of entanglement occurs due to the elongated, chain-like structures exhibited by molecules such as HA. In situations where the fluid encounters low shear rates, there is ample opportunity for the molecular chains to engage and interweave, resulting in the creation of a network-like configuration that effectively impedes the flow. The phenomenon of entanglement leads to a significant rise in the perceived viscosity of the fluid in question under the given circumstances. In essence, the variation in viscosity of synovial fluid with shear rate can be attributed to the interaction between molecular entanglement at low shear rates and shear-induced disentanglement at higher shear rates. The rheological behavior of a substance plays a crucial role in facilitating joint lubrication and preserving joint health^1^. When the flow velocity surpasses the time scale linked to random molecular motion, the ability to create organized structures that hinder relative motion becomes unattainable, leading to a subsequent decrease in flow resistance. Hence, it is crucial to measure the viscosity at various shear rates in order to precisely determine the rheological properties of the fluid^4–6^.

The Cross model is a frequently employed model for describing shear-thinning behavior in synovial fluid^1^. It offers a mathematical representation of viscosity as follows:

1 $$\begin{aligned} \eta =\dfrac{\eta _{0}}{1+(c{\dot{\gamma }})^{d}}, \end{aligned}$$

where $\eta _{0}$ denotes low shear viscosity, *c* fluids consistency is thought of as a measurement of how long it takes for the fluid to relax and *d* is the rate index, which quantifies the dependency of shear-thinning viscosity on shear rate^1,5^.

**Figure 1:**
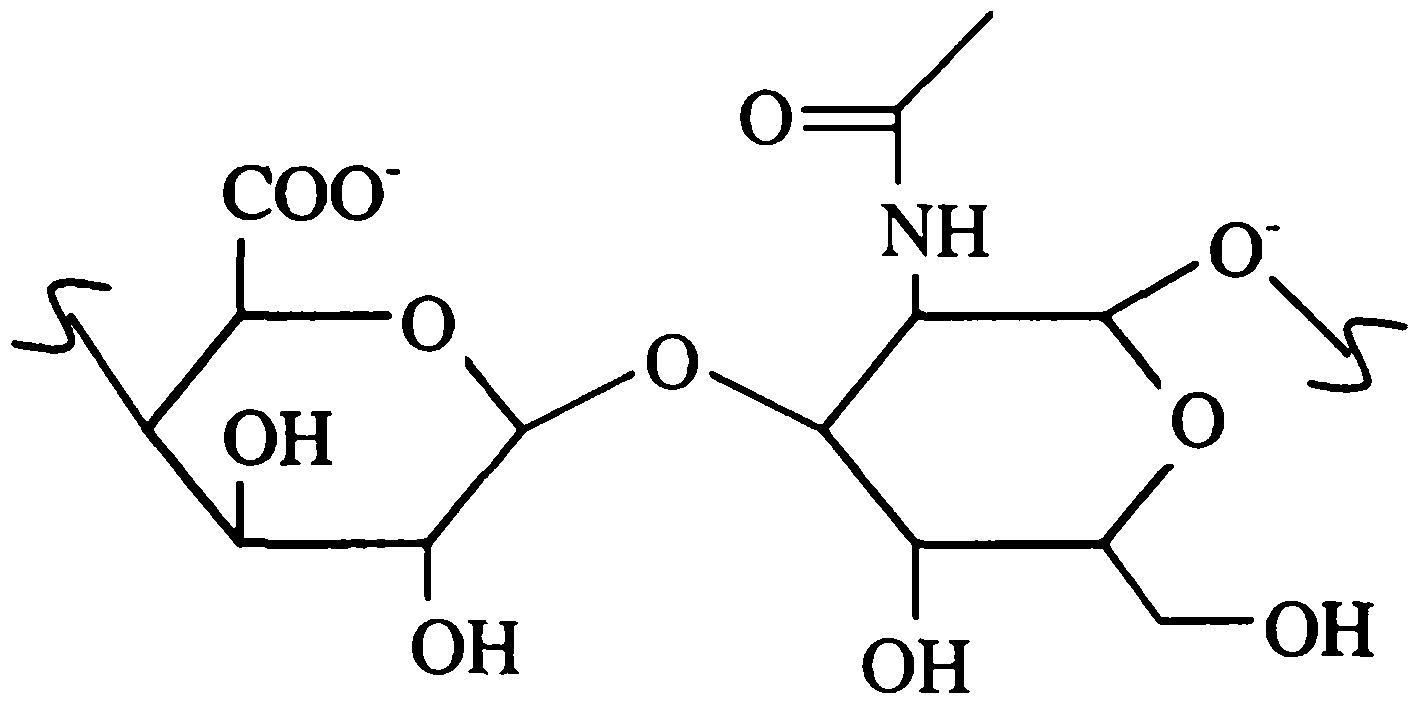
Shows an HA monomer in schematic form with n-acetyl-glucosamine, the disaccharide monomer of HA, on the right and d-glucuronic acid, the acid disaccharide, on the left^12–14^.

The shear viscosity which is steady and other relevant metrics have been recorded several times in a variety of patient groups. Rainer et al. conducted one of the most extensive research up to this point in which they analyzed more than two hundred diseased and postmortem samples however they did not describe the sizes of the individual groups^7^. Their findings which are shown below in Table 1 together with the findings of various studies are regarded as the normal ranges for postmortem and diseased SF and they are typically compatible with the discoveries made by others^7–9^. It is believed that the molecular weight and concentration of Hyaluronic Acid (HA) are the factors that decide the flow properties of SF in health and illness, the amount of HA which is contained in synovial fluid might change. HA is a kind of glycosaminoglycan that is found in high concentrations in connective tissue^10–12^. The d-glucuronic acid and n-acetyl-glucosamine units which make up the HA polymer’s repeat unit are alternated with one another, see Fig. 1. In SF, the cell which looks like fibroblast known as type B synoviocytes, are responsible for the production of HA. It is then secreted into the joint capsule, unbranched chains in SF with $10^{7}$ Da (molecular weight)^10–14^. The HA molecule has been the subject of a significant amount of study because of its peculiar physical characteristics, despite the fact that its structure seems to be rather straightforward^1,10–14^.

**Table 1:** The patient categories are outlined in the same manner as they were in the prior study

Synovial fluid’s naturally viscous characteristics					
Patients	Normal	Normal	Postmortem	OA	RA
# of Samples	2	Not Reported	Not Reported	4	2
$\eta _{0}$	>20	10–34	06–12	0.1–01	0.1
c	>10	Not Reported	40–100	1	Not Reported
d	0.75	Not Reported	0.7	0.6	0
HA	4.1 ± 1.0	1.0 ± 0.4	2.4 ± 0.9	0.9	0.7 ± 0.3
Earlier Findings	^14^	^20^	^7^	^22^	^14^

Samples of joint fluid were collected from the knees. Joints affected by rheumatoid arthritis (RA) and those affected by osteoarthritis (OA) are seen as being comparable. The measurement of viscosity is done in Pascal seconds, whereas consistency is done in (s). The rate index does not have any units. The authors indicated that more than 200 samples were investigated in total, however, they did not specify the number of samples that were included in each category^7,14,20,22^.

Since HA is an unbranched polymer, its molecular weight, and concentration may be used to describe the dissolved solution, see Fig. 1. In 1960, research was conducted in which Davies and Palfrey et al. examined three different ways of estimating the molecular weight of HA. These methods were light scattering, centrifuging, and intrinsic viscosity^15,16^. The intrinsic viscosity was the most accurate approach after fitting the data to the Mark–Houwink equation which is as follows:

2 $$\begin{aligned} \eta =0.036 M_{v}^{0.78} \end{aligned}$$

The symbol $\eta$ is used to denote the viscosity, while $M_{v}$ represents the average molecular weight in Dalton^15,16^.

However, the detrimental impact that polydispersity has on the sensitivity of centrifugation it was decided that intrinsic viscosity would be a better measurement technique to utilize. Since, the viscous and viscoelastic characteristics of HA have been described under a broad variety of different environmental circumstances^15,16^. In addition to the molecular weight and concentration of HA, the rheological behavior is typically compatible with theoretical expectations for polymer solutions^17–20^. The HA network is a shear-thinning, visco-elastic liquid when it has a molecular weight and concentration that are physiologically relevant. Therefore, the HA concentration could be able to explain a significant portion of the observed diversity in flow characteristics. However, this has not yet been shown explicitly^20,21^. The fact that healthy SF contains a larger viscosity, more HA, and a higher molecular weight than osteoarthritis (OA) or rheumatoid arthritis (RA) synovial fluid does provide evidence for the existence of a link between the two conditions^20,21^. If protein contributes to viscosity then the consequence of this would be to reduce the disparities that exist between the groups.

However, there might be additional factors contributing to this fluctuation. For instance, the vast majority of studies did not take into account, the environment of the joint may have been altered by pharmaceutical therapies. According to the findings of Dahl et al., the concentration of HA is unaffected, despite the possibility that other therapies might have an effect. For instance, viscosupplementation is an example of a pharmaceutical intervention that has the potential to alter the HA content of joint fluid^23^. Last but not least consider the diverse array of factors that may lead to osteoarthritis (OA). It is possible that various disease etiologies might result in various SF compositions. According to the findings of some preliminary research, the primary components of joint fluid seem to be the same as those of SF, protein, HA, and phospholipids^24–27^. For the measurement of the concentration of protein, HA, and phospholipids straightforward and dependable procedures have been developed, see Table 2. However, the approach that has shown to be the most accurate for measuring the molecular weight of HA has not yet been identified^26–30^.

**Table 2:** The parameters often found in synovial fluid and joint fluid, both when healthy and when pathological, are summarized in the aforementioned table

Previous research on the constituents of joint fluid			
Knee Joint/Joint Components	Healthy	Osteoarthritis	Rheumatoid Arthritis
Protein	10–30 mg/ml	24–44 mg/ml	27–63 mg/ml
Hyaluronic acid	$\sim$ 2 MDa	Mw 2.4–3.2 MDa	Mn $\sim$ 0.6 MDa
	2–4 mg/ml	0.5–1.0 mg/ml	0.1–0.9 mg/ml
Phospholipids	$\sim$ 0.1 mg/ml	0.1–0.5 mg/ml	0.4–0.8 mg/ml

When results are stable, they are reported as a 95% confidence interval (representing two standard deviations of the mean if normally distributed)^7,14,20,26–30^.

Comparing the different studies proves to be a challenging task due to the utilisation of distinct rheological models by various researchers. Dahl et al. (1985), Rainer (1996), and Seller et al. (1971) have conducted comprehensive studies and employed appropriate models for their research^23,24,30^. Due to the fact that several organizations have utilized varying ranges of shear rates, it is not possible to compute some parameters of the Cross model. One of the drawbacks of the research conducted by Rainer and colleagues is that they only looked at postmortem samples from healthy joints. Their findings probably underestimate the viscosity of synovial fluid seen in joints that are really healthy since SF may become more watery when a person passes away^22,31,32^. In order to address this issue, we provide an idea of the use of the power law model in conjunction with the stagnation point flow in order to investigate the temperature and flow behavior of synovial fluid in the knee joint under a variety of different parametric conditions. Later on, the flow at the stagnation point permits us to deliver nanofluid drugs into the SF of the knee joint. The major focus of this research is on the heat transfer and flow structure of pseudo-plastic non-Newtonian fluids flowing over a permeable flat plate in the direction of the base fluid. Energy, continuity, and momentum equations are used to describe the subject under investigation. The similarity solution is used to simplify these equations into two nonlinear ordinary differential equations (ODEs)^33–48^. The resultant ODEs then solved numerically using the software package.

In section "Steady-shear viscosity methodology evaluation", the linear scale and influence of temperature on the steady shear viscosity model are provided and evaluated. The mathematical formalism is described in section "Mathematical formulation". Section "Method of solution" then goes on to talk about the method used to solve them. Sections "Analysis" and "Conclusion" provide a study of a few dimensionless quantities and with analysis respectively.

## Steady-shear viscosity methodology evaluation

Dintenfass^14^, together with Davies^15^ conducted research on the viscosity of human SF samples in 1966, and found that the fluids associated with RA were predominantly Newtonian but the fluids associated with other disease states displayed shear-thinning and had a greater viscosity. The experiment utilized the cone-on-cone geometry with a minimal volume of SF (0.3 ml). Whereas shear-thinning was seen in bovine SF during the same year as Davies et al.^15^ in which fluid originates from the different joints. A cone and plate rheometer were used throughout all of these tests and investigations. In 1968, Palfrey et al.^16^ published findings that were comparable using an oscillating apparatus. However, they discovered that oscillatory studies were more difficult to interpret than steady-shear tests. In 1968, Ferguson et al.^17^ conducted an experiment in which they compared the SF that was extracted from diseased patients’ left and right knees. The authors of this research demonstrated an intriguing finding in that they revealed the viscosity of SF did not seem to alter for the same patient during the course of the day.

In 1976, Reimann et al.^18^ conducted an experiment in which they assessed the viscosity of 80 samples of diseased human SF using three different shear rates. In 1978, Cooke et al.^22^ conducted an experiment in which shear rates ranging from 0.1 to 1000 were used to determine the constant shear viscosity of a selection of healthy and sick human fluids. He examined one postmortem sample that was considered to be normal as well as a combined sample of another two samples. He carried out each and every one of his tests at a temperature of 21 degrees Celsius in which SF was collected from 59 individuals suffering from a variety of arthritic diseases and analyzed^23^. They made their measurements at 37 degrees Celsius using cone and plate geometries however they did not provide the shear range across which they tested. It would seem that the maximum shear rate that they used was 150 $s^{-1}$. Each sample was analyzed using a power law regression,

3 $$\begin{aligned} \sigma = \underbrace{\textbf{K}}_{=\dfrac{\eta _{0}}{\textsc {c}^{\textsc {d}}}} {\dot{\gamma }}^{n}, \end{aligned}$$

where Cross model equivalence to shear-thinning portion can be manipulated with the value of $\textbf{K}$ and *n* which is equal to $(1-d)$. Such substitution gives rise to the following equation,

4 $$\begin{aligned} \eta = \dfrac{\eta _{0}}{(c {\dot{\gamma }})^{d}}. \end{aligned}$$

When ${\dot{\gamma }}$ (shear rate) is exceeding 1 by a wide margin with $c^{-1}$ then Eq. (4) equivalence to Eq. (1). By using cytological measurements as well as the parameters $\textbf{K}$ and *n* derived from the power law model, the samples into mechanical and inflamed fluids. $\textbf{K}$ was discovered to be more than 0.03 and *n* was found to be less than 0.85 from regular SF^7,20,26–28^. These were taken from individuals who had a kind of arthritis that was not associated with any shifts in their SF. On the other hand, fluids classified as inflammatory were much less viscous and had a lower degree of shear-thinning ($\textbf{K}<0.01$, $n > 0.85$). Although they mention that there were five instances in which the results of the cytology and rheology tests were inconsistent with one another^7,20,26–28,49^. In addition, there was a category of fluids that fell somewhere in the middle ground, between those described as inflamed and mechanical.

Research on the viscosity of normal SF was the subject of a number of research that were published in the 1980s. Two separate rheometers were utilized to cover the range of shear rates from 0.001 s$^{-1}$ to 1000 s$^{-1}$ in brief reviews published in 1987 and 1996, respectively^5,16,22,23,26^. The steady-shear properties of SF may be determined using a variety of experimental equipment some of which have already been utilized to make a determination. Since then, there have been developments in technology that enable devices to measure rheological characteristics across up to three orders of magnitude of shear rates. However, describing flow properties over a greater range requires the use of many devices. Because the devices should provide comparable outcomes after being properly calibrated, the distinctions between them are not explored in more depth here. In the context of knee joint, the apparatus is used to assess the flow characteristics of joint fluid has a double cylinder shape that may be approximately represented as two parallel flat plates or flow over a stretched surface with various base fluids^49–55^.

### Viscoelasticity on a linear scale

In addition to the non-Newtonian behavior, it has been shown that SF also has elastic characteristics (i.e. energy storage). The behavior of visco-elastic materials under enormous deformations that may be significant in vivo is difficult to investigate using computer methods because of the complexity involved. The behavior of a viscoelastic material, on the other hand, is independent of the amplitude of the motion it is subjected to for movements with sufficiently small amplitudes and it can be defined by a few parameters^56,57^.

Researchers have started to define complicated fluids in the linear viscoelastic range even if meaningful motion may occur at greater deformations which is important. The physical entanglement that occurs among long molecules is one of the sources of viscoelastic motion in liquids. The network does not get disentangled even when it is exposed to deformation of a modest amplitude particularly when this deformation occurs across brief time periods. Instead, the chains are stretched and compressed which results in the storage of energy in a manner similar to that of a solid subjected to elastic deformation. However, molecular disentanglement is triggered by processes that include larger movements and longer time periods^2,25,26,57^. Therefore, the most obvious manifestation of elastic behavior in liquids is seen in motions with low amplitudes and high velocities. This circumstance when it occurs in a human joint, is analogous to activities like running or leaping (3 Hz). Other movements such as standing might have substantially longer characteristic periods than walking which happens at a frequency closer to one hertz (1 Hz). Some people feel that the viscoelastic qualities of SF help to sustain and preserve joint tissue even when the joint is subjected to high-velocity motion^2,25,26,57^.

### The effects of temperature

The temperature has an effect on the viscosity of SF which was something that was not really touched on in the previous discussion. The rheological behavior of various polymer solutions is seen to be dependent on temperature in the same way as it is dependent on time. This observation is supported by some molecular evidence^58^. The link between temperature and viscosity in joint fluid was observed across the range of 25–40$\,^{\circ }$C. Viscosity decreases as temperature rises which was shown to be the case throughout this temperature range^59–63^. In line with the finding, Webb et al.^28^ observed within the same body of research that investigated the piezo viscous effect in which viscosity dropped by a factor of 2.5 to 5 times between the temperatures of 5 and 35 degrees Celsius. The majority of researchers working in this area have examined the rheology of SF at temperatures that are somewhat close to $25\,^{\circ }$C. Some researchers have stated that SF evaporates fast at physiological temperatures although protein denaturation and precipitation proved a larger concern than evaporation^64,65^. In addition, it was difficult to determine elastic characteristics at higher temperatures, most likely because of the temporal temperature superposition effects. As a result, it has been determined that evaluating these qualities at a temperature of $25\,^{\circ }$C is the most acceptable method for making easy comparisons between samples and with the work done by others in the past^53–55^.

## Mathematical formulation

**Figure 2:**
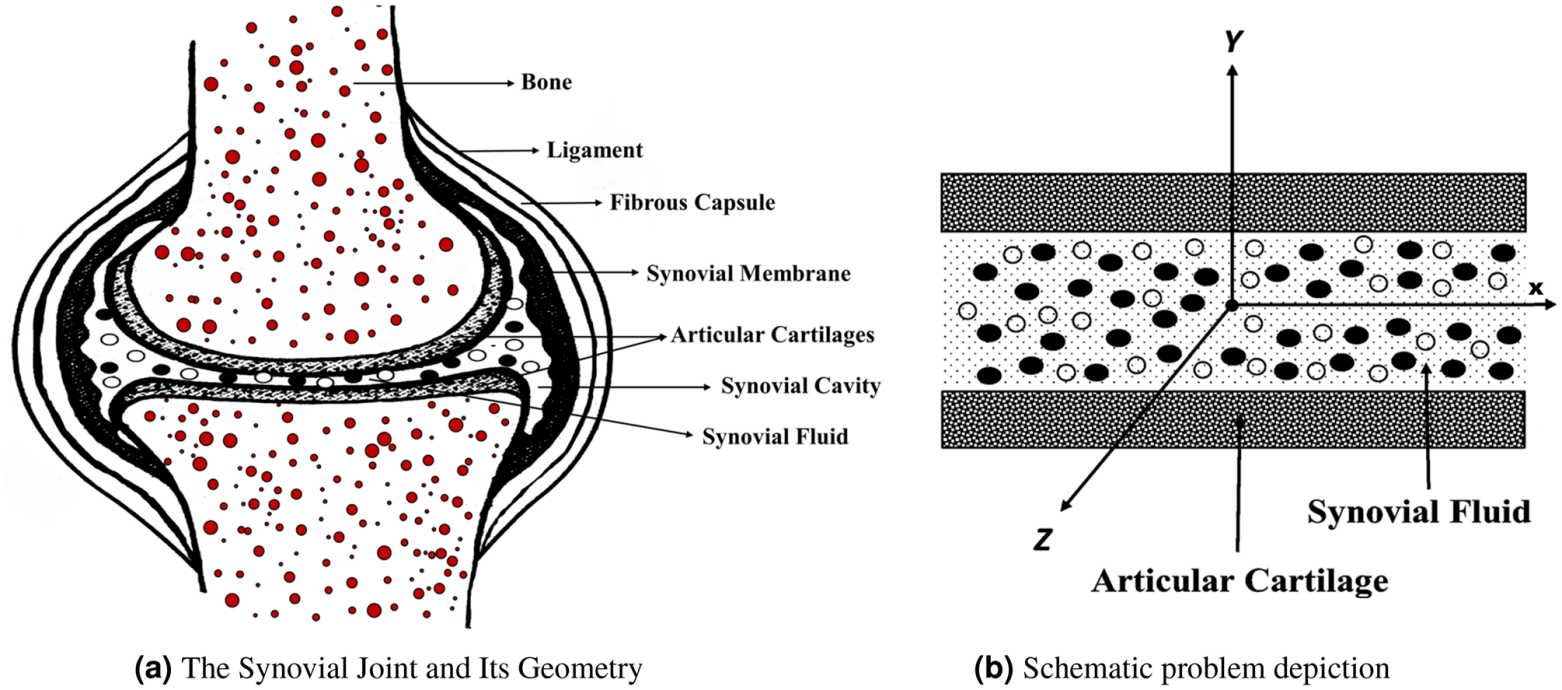
Illustrates the Synovial Joint (**a**) and provides a schematic representation (**b**) of the research problem^66^.

The flow in the boundary layer is two-dimensional and in a steady state, the flat plate is unmoving and porous. Both the velocity and temperature of the free stream are equal to $U_{\infty }$ and $T_{\infty }$. The base fluid is a non-Newtonian fluid that has a pseudo-plastic consistency. Experiments have shown that the thermophysical properties of CMC/water (base fluid (bf)) and those of water are quite similar to one another. The model takes into consideration a flow regime that is laminar and incompressible, see Fig. 2. In addition, one of the presumptions is that there is no transition between fluid phases, and the condition of their thermal equilibrium is stable. The continuity equation, the momentum equation, and the energy equation are the governing equations. Also, experiments on mechanical degradation imply that HA macromolecules are present, which provides strong evidence for the hypothesis that the stagnation point extensional flow field contains almost the entire extension of the high molecular weight component. When taken as a whole, the findings point to a potential approach for analyzing the HA in synovial fluid which is used to assess the viability of this notion.

In the governing system, $u^{*}$ and $v^{*}$ presents flow velocity in *x* and *y* directions respectively. The power-law number is indicated by the letter *n* in the exponent, which is connected to the base fluid. Both Newtonian fluids and pseudo-plastic fluids have *n* values that are either 1 or between 0 and 1, depending on the kind of fluids. Additionally, the value of *n* for dilatant fluids is greater than 1. The mass transfer velocity, denoted here by $V_{w}$, is something that ought to be thought of as being next to the plate. In the case of an impermeable plate, $V_{w}$ equals 0. When there is blowing or injection present, $V_{w}$ has a positive value, but when there is suction present, it has a negative value. The plate becomes heated from the bottom up by a process known as convection, in which a fluid whose temperature is equal to $T_{f}$ and whose heat transfer coefficient is $h_{f}$ is responsible for the heating^67,68^. The dimensional governing equations for the boundary layer may be stated as follows;

5 $$\begin{aligned} \frac{\partial u^{*}}{\partial x}+\frac{\partial v^{*}}{\partial y}&=0, \end{aligned}$$

6 $$\begin{aligned} u^{*}\frac{\partial u^{*}}{\partial x}+v^{*}\frac{\partial u^{*}}{\partial y}&=\frac{\mu _{bf}}{\rho _{bf}}\frac{\partial }{\partial y}\left( |\frac{\partial u^{*}}{\partial y}|^{n-1}\frac{\partial u^{*}}{\partial y}\right) -U_{\infty }\frac{\partial U_{\infty }}{\partial x}-\frac{\sigma _{bf}B_{0}^{2}}{\rho _{bf}}(u^{*}-U_{\infty }) \end{aligned}$$

7 $$\begin{aligned} u^{*}\frac{\partial T^{*}}{\partial x}+v^{*}\frac{\partial T^{*}}{\partial y}&=\alpha _{bf}\frac{\partial }{\partial y}\left( |\frac{\partial T^{*}}{\partial y}|^{n-1}\frac{\partial T^{*}}{\partial y}\right) . \end{aligned}$$

Boundary conditions are given as:

8 $$\begin{aligned} &u^{*}\rightarrow U_{\infty }\ \ \ as \ \ \ y \rightarrow \infty , \end{aligned}$$

9 $$\begin{aligned} &u^{*}=0, \ \ \ v^{*}=V_{W}(x)\ \ \ at\ \ \ y=0. \end{aligned}$$

10 $$\begin{aligned} &-k_{bf}\frac{\partial T^{*}}{\partial y}=h_{f}(T_{f}-T_{w})\ \ \ at\ \ \ y=0, \end{aligned}$$

11 $$\begin{aligned} &T^{*}\rightarrow T_{\infty }\ \ \ as \ \ \ y \rightarrow \infty . \end{aligned}$$

For the present study, CMC/water has been considered as the base fluid (bf) with $Pr=6.2$. According to a review article by Oztop et al.^54^, the thermo-physical properties are as follows:

12 $$\begin{aligned} &\alpha _{bf}=\frac{k_{bf}}{(\rho C_{p})_{bf}}, \ \ \ \mu _{bf}=\frac{u}{(1-\varphi )^{2.5}}, \end{aligned}$$

13 $$\begin{aligned} &\frac{k_{bf}}{k_{f}}=\frac{(k_{s}+2k_{f})-2\varphi (k_{f}-k_{s})}{(k_{s}+2k_{f})+\varphi (k_{f}+k_{s})}, \end{aligned}$$

14 $$\begin{aligned} &(\rho C_{p})_{bf}=(1-\varphi )(\rho C_{p})_{f}+\varphi (\rho C_{p}), \ \ \ \rho _{bf}=(1-\varphi )\rho _{f}+\varphi \rho _{s}. \end{aligned}$$

## Method of solution

We provide the following similarity transformations to facilitate the mathematical analysis of our research.

15 $$\begin{aligned} &\psi =(U_{\infty }^{2n-1}vf_{x})^{\frac{1}{n+1}}f(\eta ), \ \ \ u^{*}=\frac{\partial \psi }{\partial y}, \ \ \ v^{*}=-\frac{\partial \psi }{\partial x}, \end{aligned}$$

16 $$\begin{aligned} &\eta =\left( \frac{U_{\infty }^{2-n}}{v_{f}x}\right) ^\frac{1}{n+1}y, \ \ \ \theta =\frac{T^{*}-T_{\infty }}{T_{f}-T_{\infty }} \end{aligned}$$

Solving Eqs. (5) to (12), we get the following ordinary differential systems (ODEs);

17 $$\begin{aligned} &\frac{1}{A_{4}A_{1}}(|f''|^{n-1}f'')'+\frac{1}{n+1}f''f-M^{2}\frac{\sigma _{bf}}{\sigma _{f}}(f'-1)-\epsilon ^{2}=0, \end{aligned}$$

18 $$\begin{aligned} &-\frac{A_{3}}{Pr_{f}A_{2}}(|f''|^{n-1}\theta ')'+\frac{1}{n+1}f\theta '=0, \end{aligned}$$

where

19 $$\begin{aligned} \frac{\sigma _{bf}}{\sigma _{f}}=1+\frac{3\varphi \left( \frac{\sigma _{s}}{\sigma _{f}}\right) -1}{\left( \frac{\sigma _{s}}{\sigma _{f}}+2\right) -\varphi \left( \frac{\sigma _{s}}{\sigma _{f}}\right) -1}, \ \ \ M=\sqrt{(\frac{xB_{0}^{2}}{\rho _{bf}})}\,\ \end{aligned}$$

Boundary conditions are transformed which are as follows;

20 $$\begin{aligned} &f(0)=f_{w},\ \ \ f'(0)=0,\ \ \ f'(\infty )=1, \end{aligned}$$

21 $$\begin{aligned} &\theta '(0)=A_{3}a(1-\theta (0)),\ \ \ \theta (\infty )=0. \end{aligned}$$

## Dimensional analysis

The quantities of physical interest in this work are the local Skin friction coefficient $C_{f}$ and the local Nusselt number $N_{u_{x}}$ which are defined as;

22 $$\begin{aligned} C_{f_{x}}=-\frac{2\tau _{w}}{\rho _{f}U^{2}},\ \ \ N_{u_{x}}=\frac{xq_{w}}{k_{f}(T_{w}-T_{\infty })}, \end{aligned}$$

where

23 $$\begin{aligned} \tau _{w}=\mu _{bf}\left( |\frac{\partial u^{*}}{\partial y}|^{n-1}\frac{\partial u^{*}}{\partial y}\right) _{y=0},\ \ \ q_{w}=-k_{bf}\left( \frac{\partial T^{*}}{\partial y}\right) _{y=0}. \end{aligned}$$

By employing similarity transformation to Eqs. (22) and (23), we get the following results;

24 $$\begin{aligned} &Cf_{x}Re_{x}^{\frac{1}{n+1}}=-2\frac{\mu _{bf}}{\mu _{f}}f''(0)|f''(0)|^{n-1}, \end{aligned}$$

25 $$\begin{aligned} &Nu_{x}Re_{x}^{\frac{-1}{n+1}}=-\frac{k_{bf}}{k_{f}}\theta '(0). \end{aligned}$$

## Analysis

We explore the flow of both Newtonian and non-Newtonian fluids that could be utilized to model the synovial fluid flow. The SF flow equations are not amenable to easy mathematical investigation since the real geometry of the area where the flow occurs is fairly complicated. Thus, analyzing the flow in a simple geometry can help to explain the model’s efficacy and assess the SF models. Therefore, the complex interaction between the permeability of cartilage, the stagnation point, and the magnetic field as it relates to temperature distribution in a synovial joint has been analyzed using a more straightforward mathematical model. The heating caused by friction might potentially have an effect on the physical qualities of the articular surface as well as the properties of the synovial fluid.

### Analysis with respect to power law index

**Figure 3:**
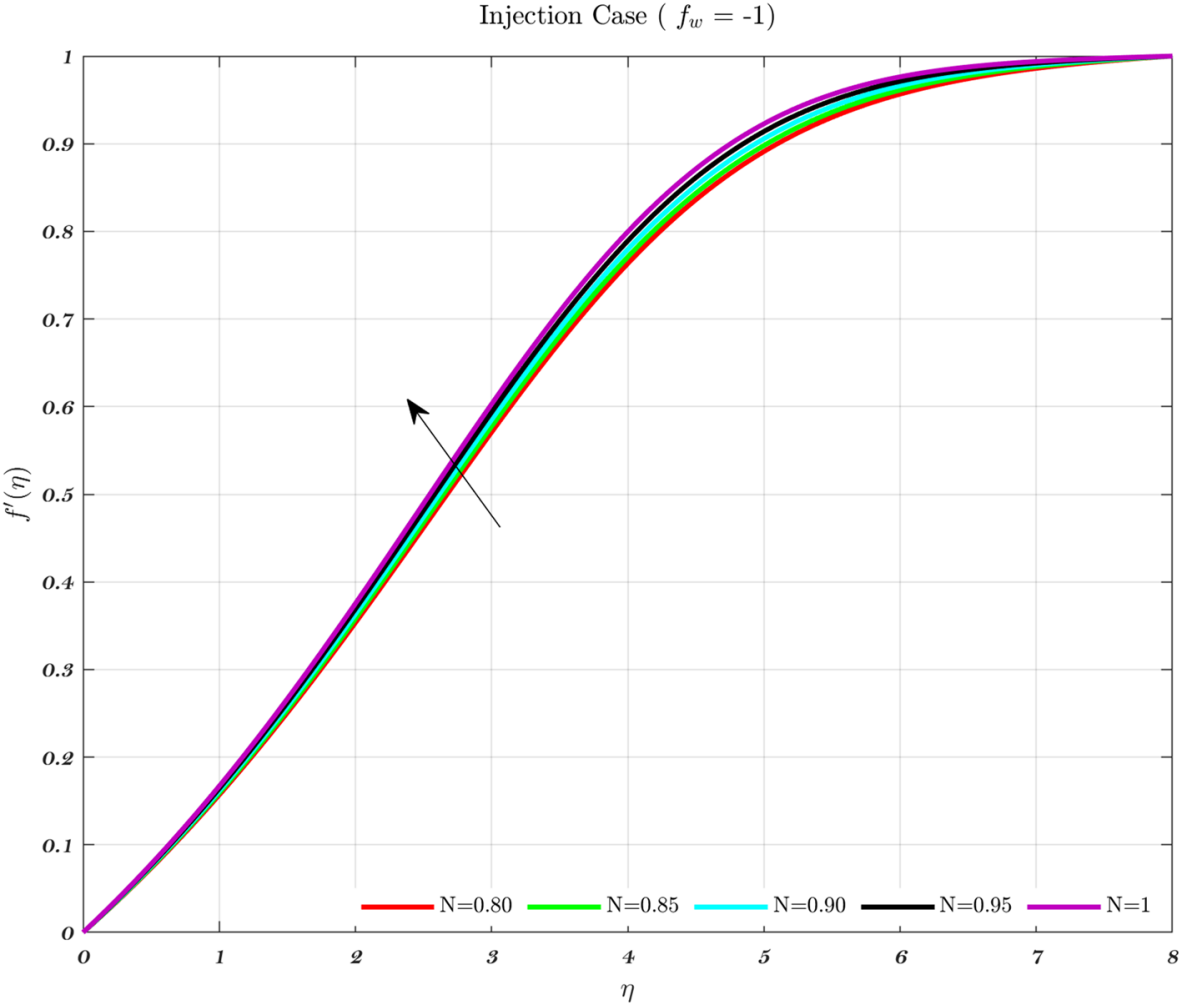
The influence of various values of *n* on the non-dimensionalized velocity in the context of the Injection case for both flows.

**Figure 4:**
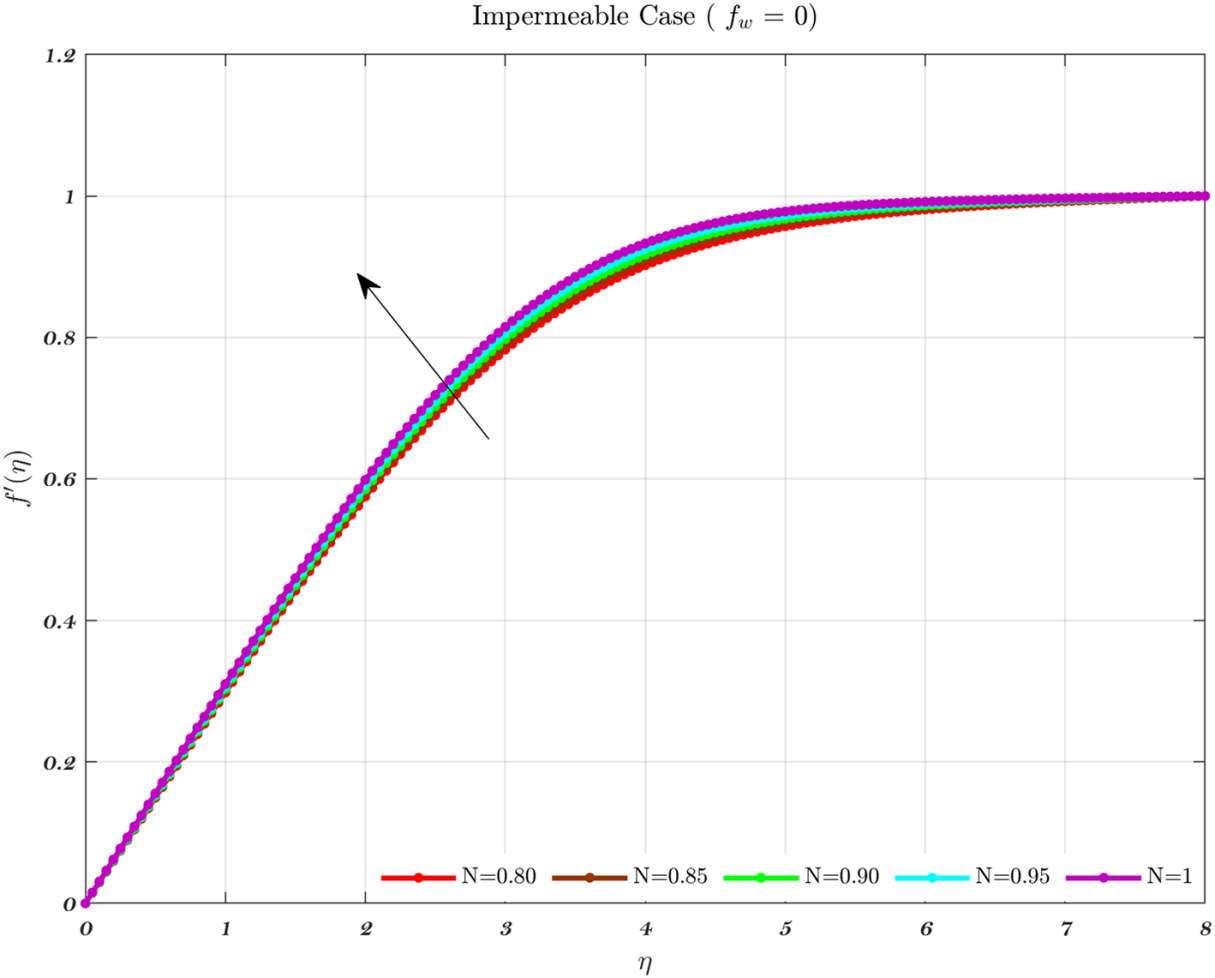
The influence of various values of *n* on the non-dimensionalized velocity in the context of the Impermeable surface for both flows.

**Figure 5:**
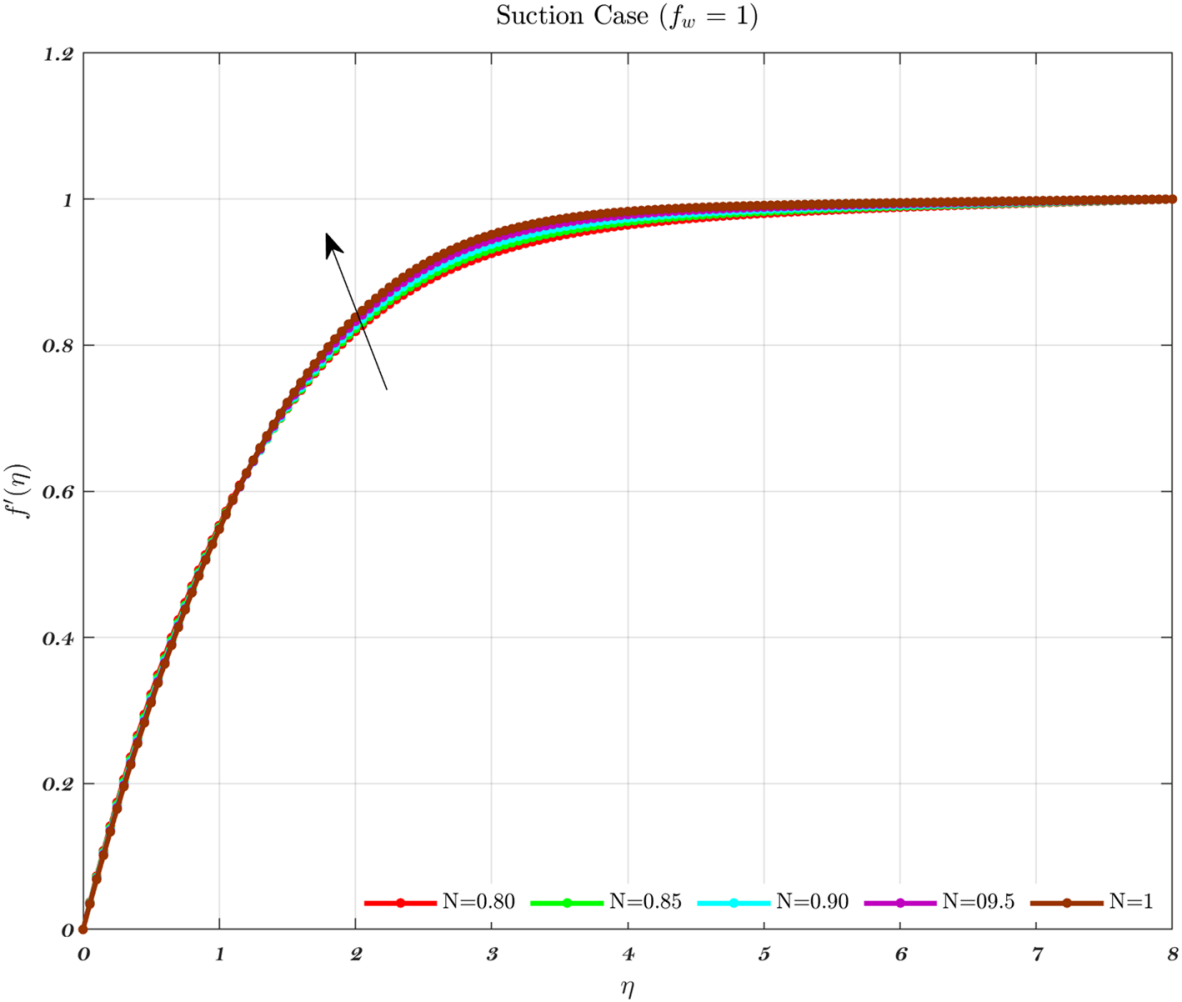
The influence of various values of *n* on the non-dimensionalized velocity in the context of the Suction case for both flows.

The impact of the Newtonian and Non-Newtonian fluids for the three different $f_{w}$ values (impermeable surface, suction, and injection) has been depicted in Figs. 3, 4 and 5 with connection to the following equation,

26 $$\begin{aligned} &V_{W}(x)=\frac{-1}{n+1}\left( \frac{v_{f} U_{\infty }^{2n-1}}{x^{n}}\right) ^{\frac{1}{n+1}}f_{w}, \end{aligned}$$

27 $$\begin{aligned} &h_{f}=c x^{\frac{-1}{n+1}}, \end{aligned}$$

where $f_{w}$ and *c* are constant numbers. Hence, the utilization of suction or impermeable surfaces leads to an augmentation in the quantity of both Newtonian and Non-Newtonian fluids, resulting in an elevation of fluid velocity. However, the converse effect occurs during injection phenomena. Also, the boundary layer thickness is decreased by utilizing Newtonian fluid for suction and an impermeable surface, but the velocity gradient is unaffected while Non-Newtonian fluids have a greater velocity gradient across the surface during injection, and such difference is accentuated by increasing the fluid concentration. The impact that permeability has on the velocity profile of both Newtonian and non-Newtonian fluids is seen in the Figs. 3, 4, and 5.

Suction has the effect of slowing down the expansion of the boundary layer, which corresponds with the observation, that the velocity slows down in direct proportion to the suction parameter^20,23,25^. Nevertheless, during injection, the boundary layer area expands, which is an indicator that injection generates greater fluid diffusion and extends the boundary layer. A shift in the velocity profile close to the walls is produced when *n* is varied from its default value. The shear-thinning and chemically thickening viscosity are to blame for this peculiar behavior seen by the velocity profile. It is important to note that although the velocity profile for $f_{w}=1$ comes very close to being linear (Newtonian), it is still considered to be a nonlinear profile. As the value grows, the velocity approaches a steady state in the central part of the domain, while the gradients get more severe as approach the plates^69^. The flow changes gradually while maintaining a nearly constant viscosity. The rise in the concentration of HA has the greatest effect on the domain’s center, and as a consequence, the viscosity around the center of the domain has grown quite high, whilst it is relatively low near the domain’s boundaries^69^.

**Figure 6:**
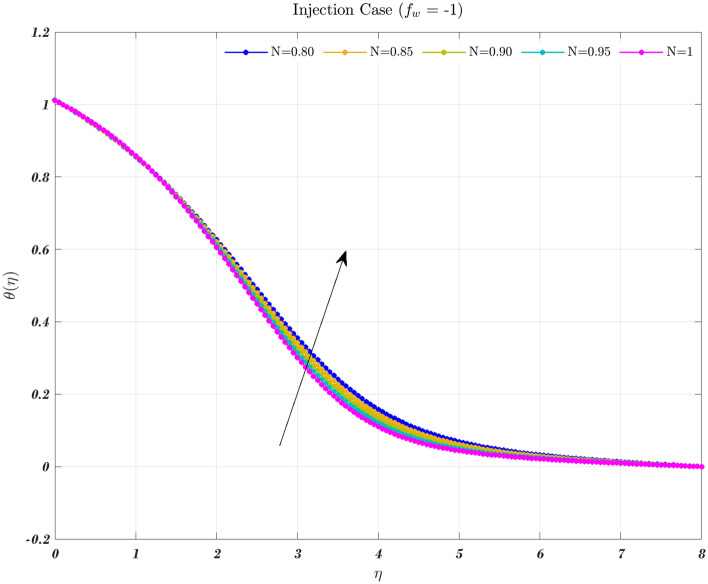
The non-dimensionalized temperature in the Injection case for both flows is influenced by different values of *n* by considering $\epsilon =0.01$, $a=0.1$, and $\varphi =1\%$.

**Figure 7:**
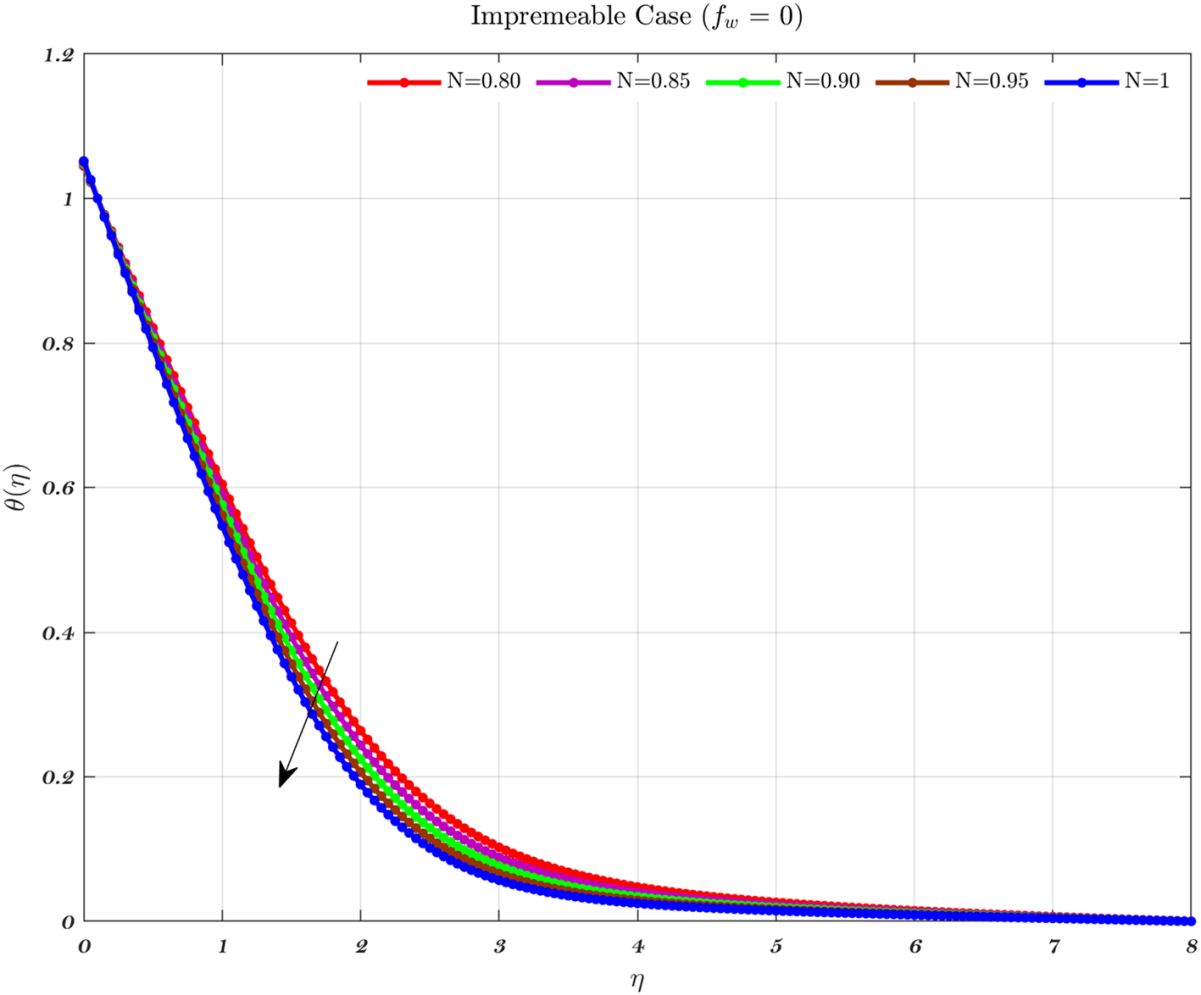
The non-dimensionalized temperature in the Impermeable surface case for both flows is influenced by different values of *n* by considering $\epsilon =0.01$, $a=0.1$, and $\varphi =1\%$.

**Figure 8:**
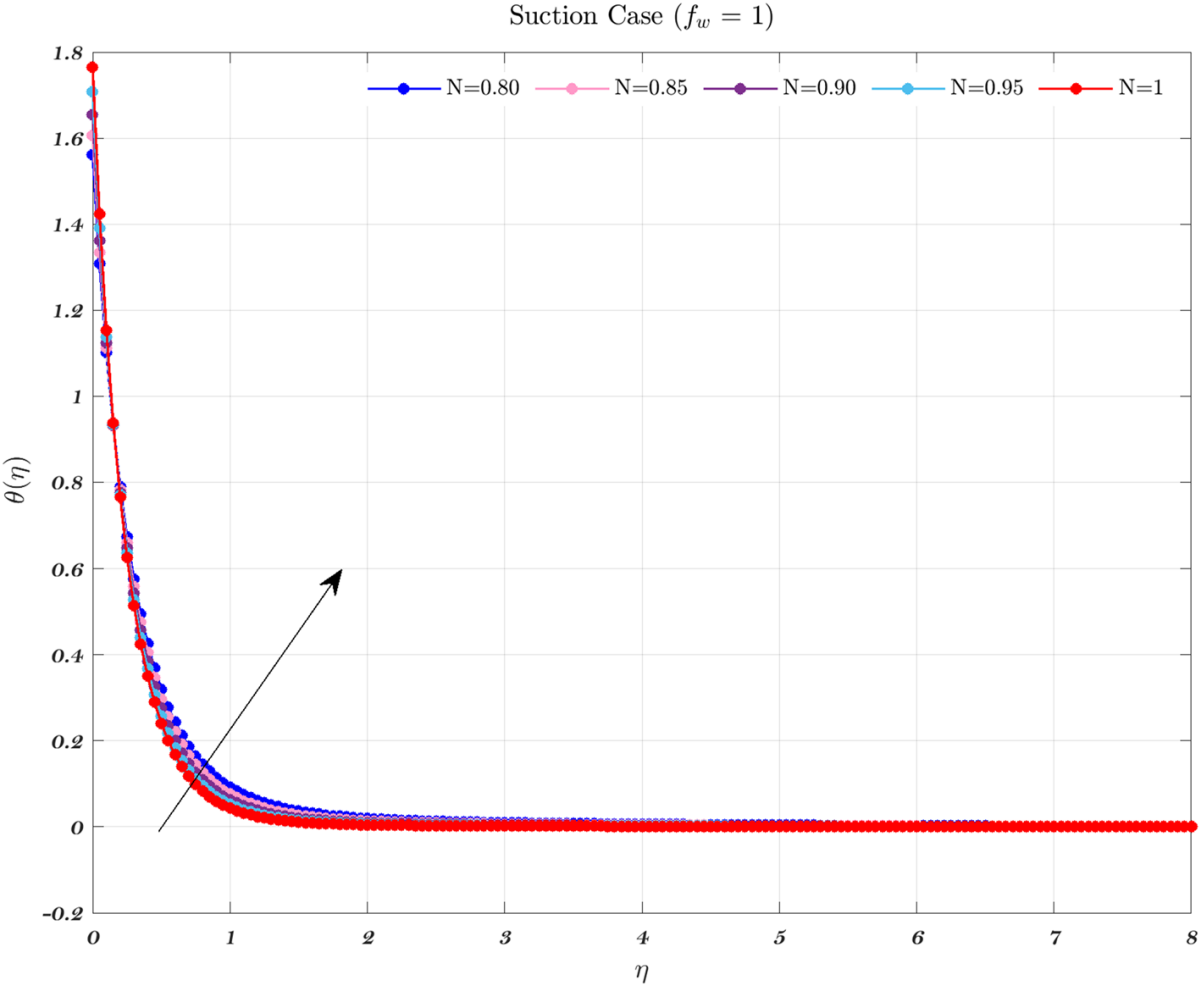
The non-dimensionalized temperature in the Suction case for both flows is influenced by different values of *n* by considering $\epsilon =0.01$, $a=0.1$, and $\varphi =1\%$.

Temperature profiles for an impermeable surface, suction, and injection are shown in Figs. 6, 7, and 8. It is observed that the dimensionless temperature decreases initially for the suction case, which involves Newtonian to non-Newtonian fluids, and for the impermeable surface case, the temperature eventually rises. Therefore, decremental temperature to the impermeable surface is combined with a non-Newtonian fluid henceforth the effect of augmentation is taken into account. When it is subjected to suction, the fluid concentration does not have a significant effect^70^.

In every instance, the thickness of the boundary layer is reduced when a non-Newtonian fluid is used. Physically, a rise in temperature of the synovial fluid because of the viscous nature of the joint tissues, causes a portion of the input mechanical energy to be dissipated as heat^69^. Thus, high temperature near the surface of the articular cartilage drops as it moves toward the contact between the bone and cartilage. This is because of the fact that friction causes a localized increase in temperature at the cartilage surface, therefore the temperature of the fluid likewise rises^13–15,18^. In the process of inhibition, the fluid is absorbed into the cartilage due to heat therefore, cools down as moves closer to the bone cartilage interface. According to Figs. 6, 7, and 8, it can be seen that the temperature drops as one moves further and deeper into the cartilage.

Hence, the Impact of different permeability on non-dimensionalized velocity and temperature profiles along flow characteristics of joints over a surface with various fluids can be seen in Figs. 3, 4, 5, 6, 7 and 8.

### Analysis with respect to magnetic field

**Figure 9:**
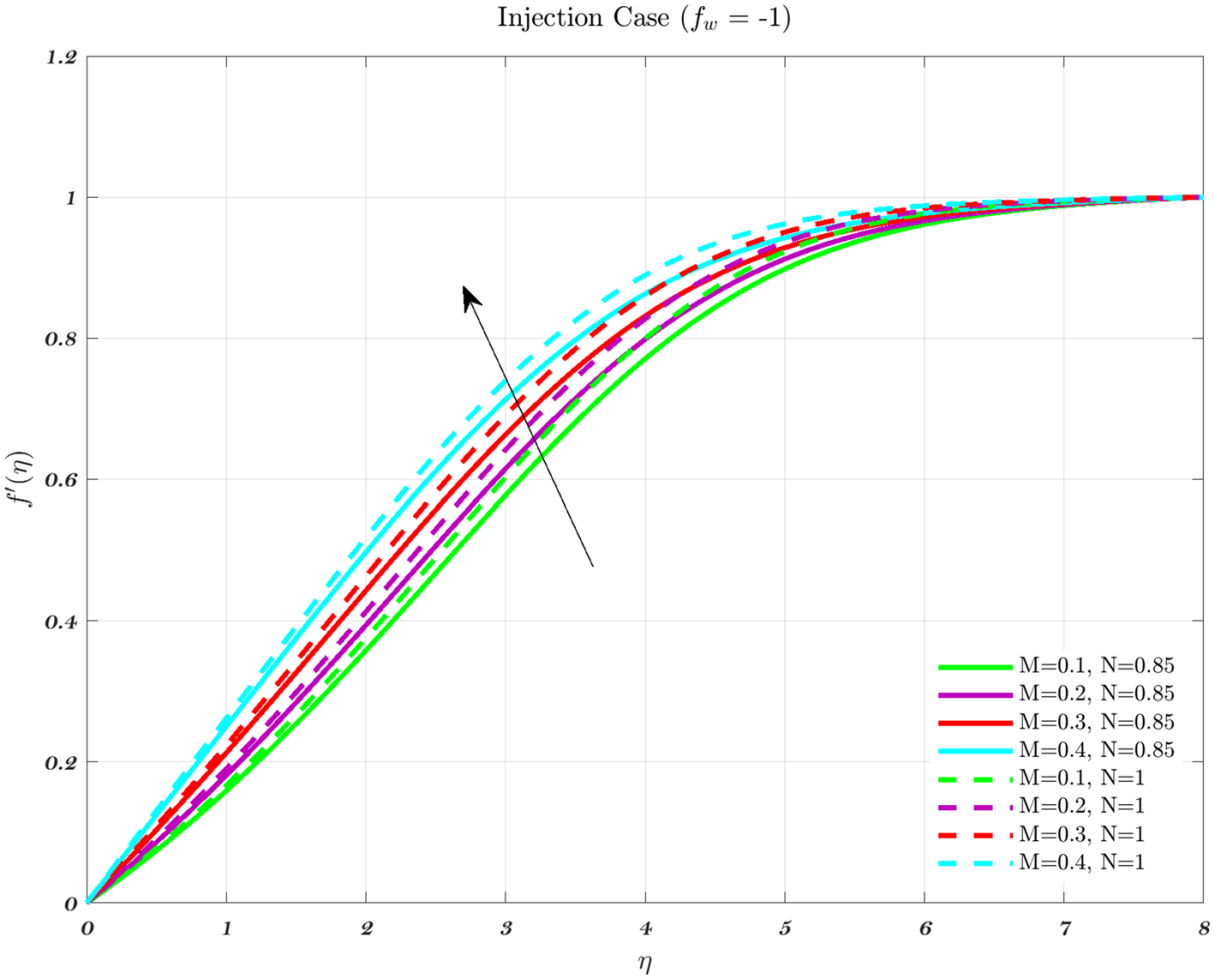
In the context of Injection, the non-dimensional velocity for both fluids is determined by considering different values of the magnetic parameter *M*.

**Figure 10:**
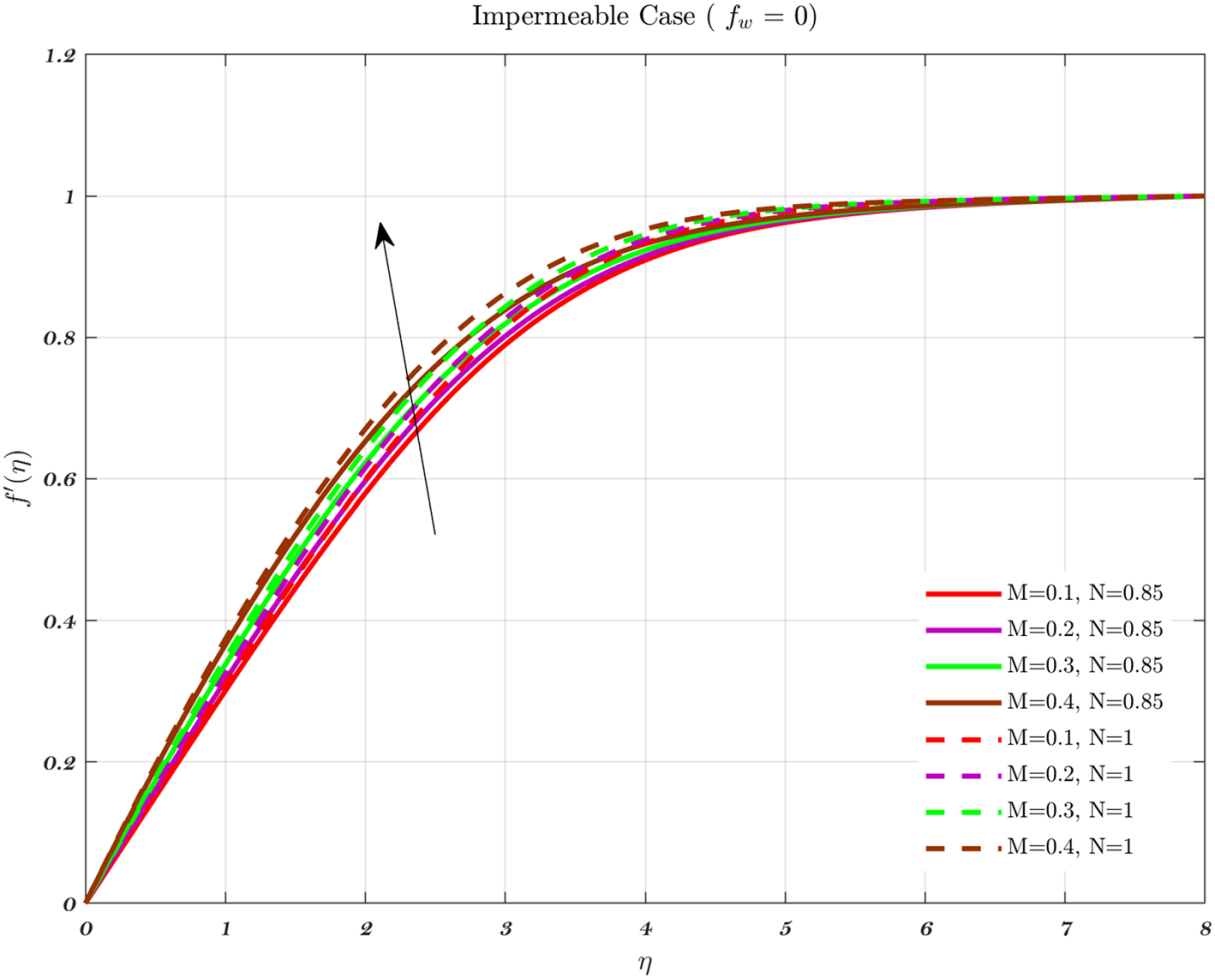
In the context of Impermeable surface, the non-dimensional velocity for both fluids is determined by considering different values of the magnetic parameter *M*.

**Figure 11:**
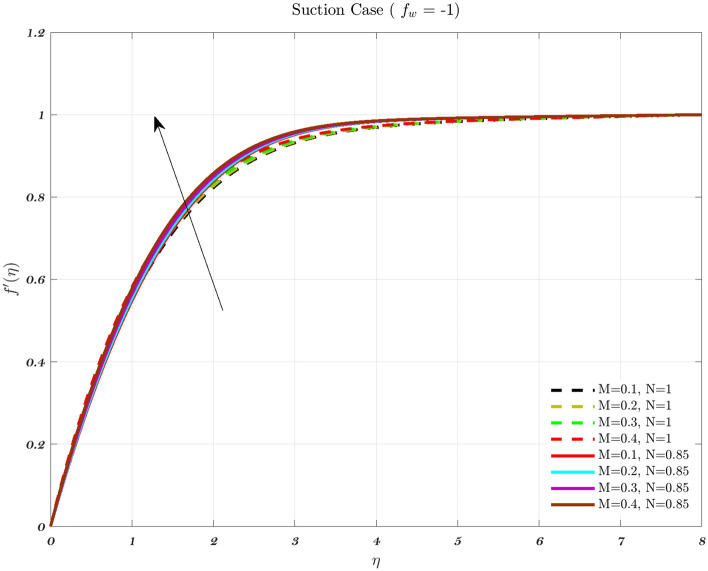
In the context of Suction, the non-dimensional velocity for both fluids is determined by considering different values of the magnetic parameter *M*.

Figures 9, 10 and 11 indicate the impact of the governing parameter such as the magnetic field of the dimensionless velocity for the features of suction, injection, and impermeability, respectively for both Newtonian and non-Newtonian fluids. This represents a physical scenario with changeable free stream conditions for both kinds of fluids. The thickness of the boundary layer will decrease in response to an increase in the intensity of the magnetic field, as measured by the Lorentz force associated with the magnetic field. The viscosity effects on the fluid are canceled out by the magnetic field, which causes the fluid to accelerate in the opposite direction from what it would have done without the magnetic field^43,44,71^. Therefore, the velocity of the synovial fluid increases as the parameter *M* rises for both Newtonian and non-Newtonian fluids in the case of knee joints. When there is a greater distance between the bones of a joint, the axial velocity slows down. As the power-law index is increased, there is a corresponding rise in the mean concentration distribution^72^.

**Figure 12:**
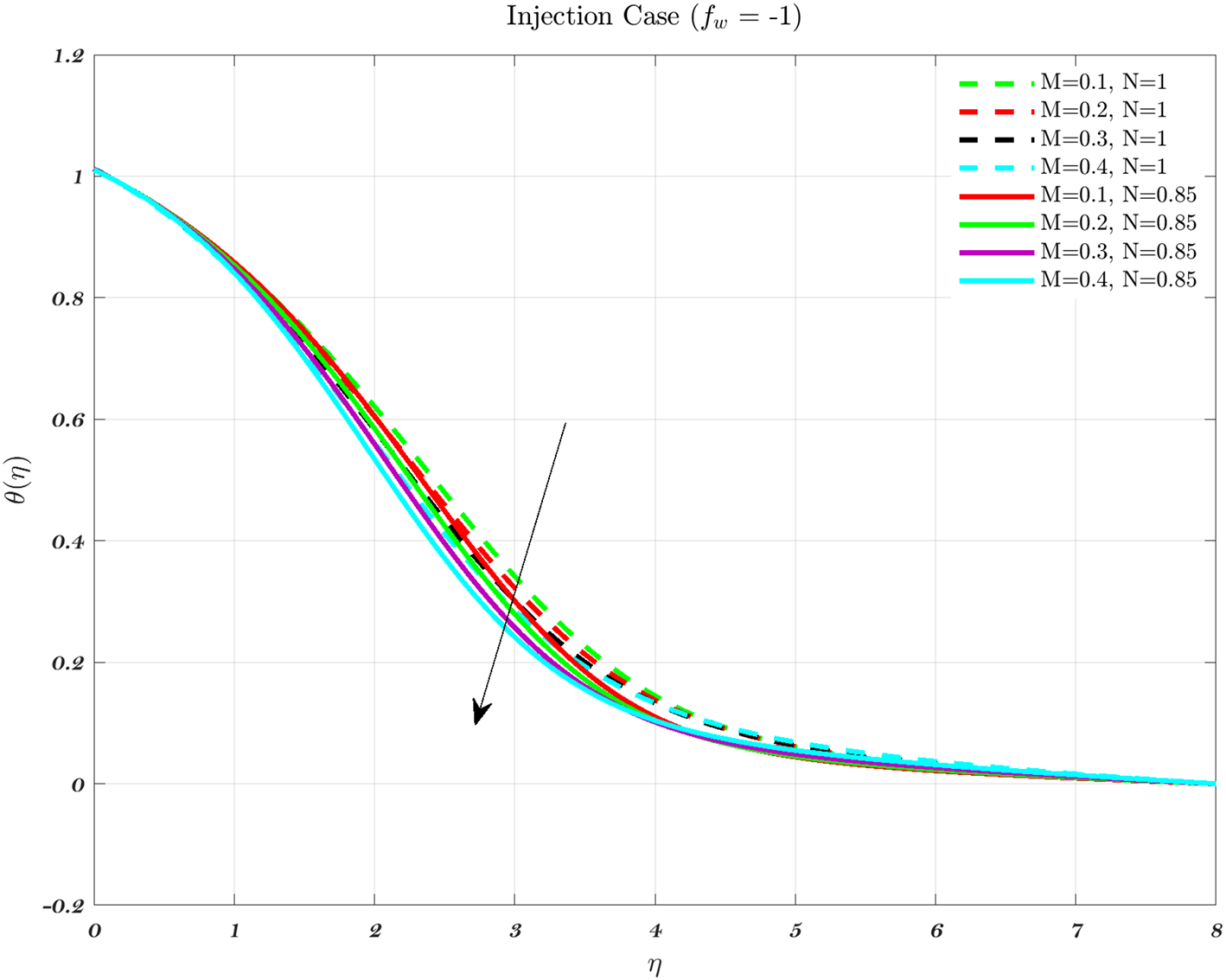
Considering different magnetic parameter *M* values determines the non-dimensional temperature for both fluids for Injection.

**Figure 13:**
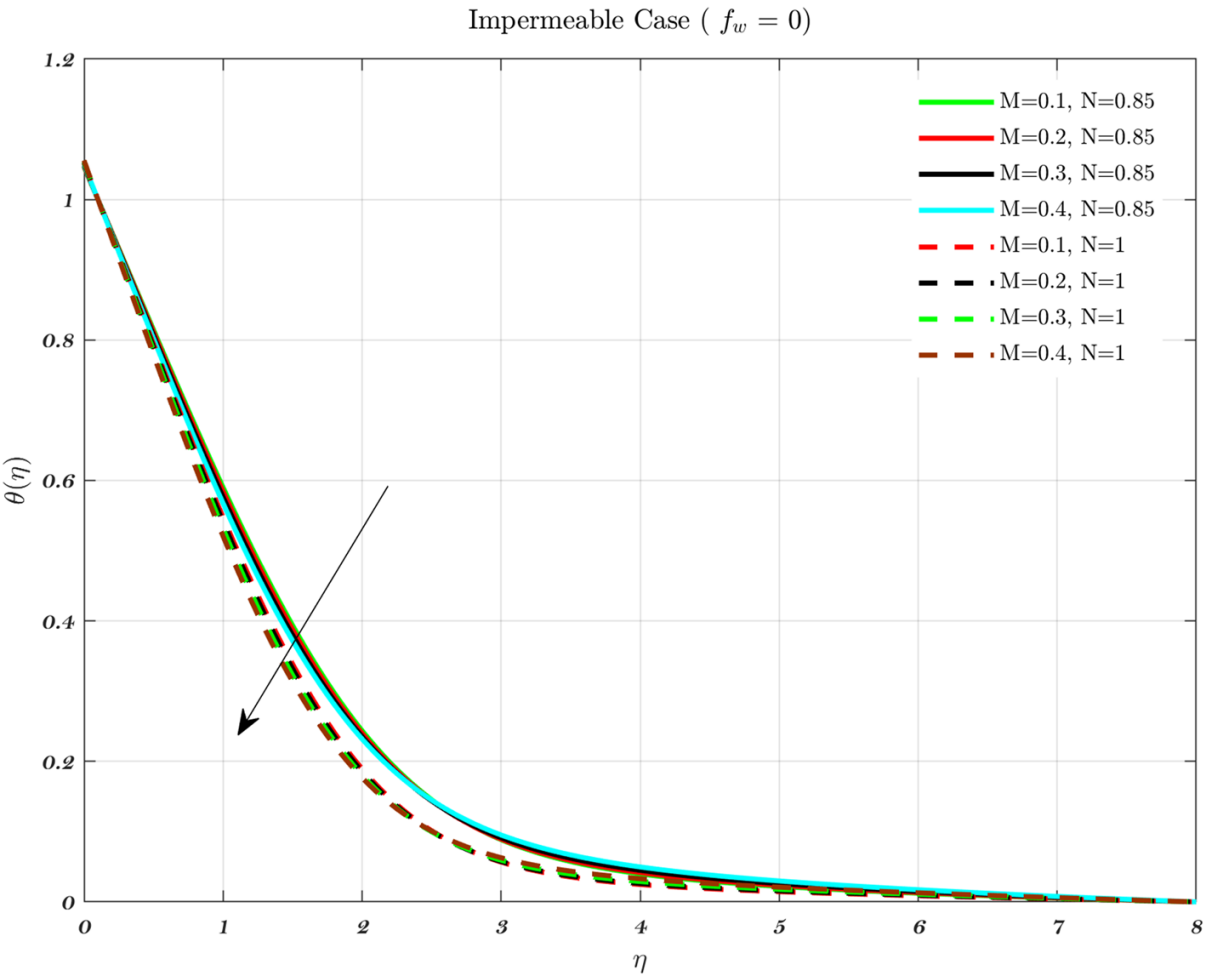
Considering different magnetic parameter *M* values determines the non-dimensional temperature for both fluids for an Impermeable surface.

**Figure 14:**
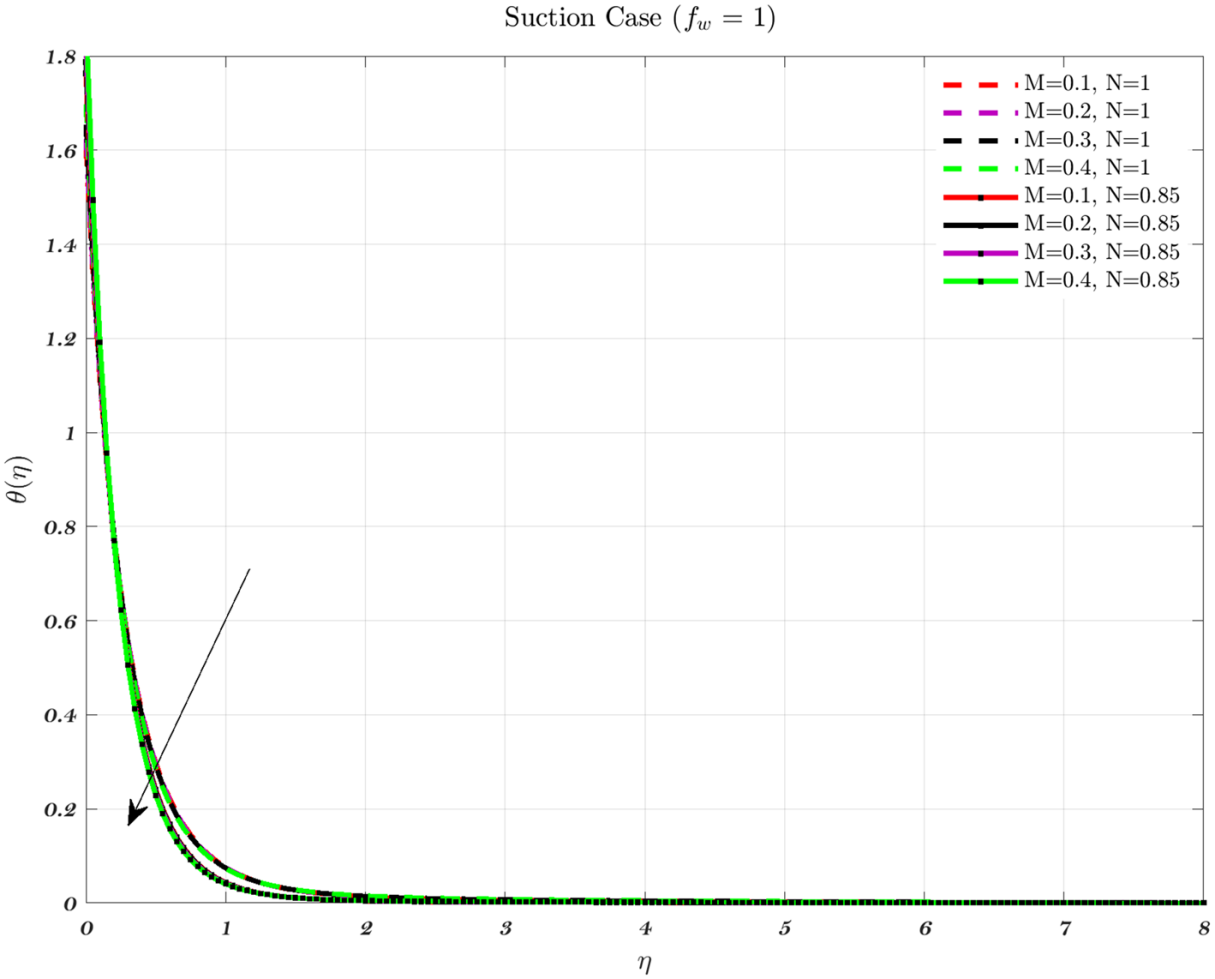
Considering different magnetic parameter *M* values determines the non-dimensional temperature for both fluids for Suction.

Figures 12, 13 and 14 show the temperature distribution for various values of the magnetic interaction parameter for the Newtonian and non-Newtonian fluids in the knee joints subject to various permeability impacts on flow conditions. Increasing the levels of the magnetic field helps to suppress the temperature distribution for both of the fluids. In addition, the thermal boundary layer thickness decreases with increasing *M* in the presence of varied free stream conditions henceforth the interaction between the applied magnetic field and the fluid particles, the temperature distribution improves with higher levels of the magnetic field for both the fluids^48,72^. The thickness of the thermal boundary layer grows in proportion to the magnetic field in the synovial fluid scenario for the Newtonian and non-Newtonian fluids. It has been noticed that the passage of fluid into or out of the cartilage is impeded in some diseases and/or aged synovial joints, leading to an increase in temperature because of the fact that the temperature rises^22,31,49^.

When the values of the parameters are changed from their usual levels, a spike in temperature is observed in aged and sick joints^2,3,5^. These numbers allude to the effects of aging as well as disorders that might impact the degradation of synovial fluid as well as cartilage. Even if they are not very noteworthy, increases in temperature result in a reduction in the viscosity of the synovial fluid^2,3,5^. It is projected that the global average temperature would increase by little more than 1 degree Celsius, and there would be some regionally heightened temperature gradients. Generally, the temperature varies greatly depending on the articulate gap such that the synovial fluid closure to the articular cartilage^3,5^. Thus, the peak temperature is roughly the same as the temperature of the cartilage surface. Therefore, an increase in knee contact forces causes a higher amount of heat to be generated. Hence, a heavier patient will have more knee contact during a given activity, then it stands to reason that would create more heat, and lead to rising temperature both within and around the knee joint^3,5,12,15^.

### Analysis with respect to stagnation point

**Figure 15:**
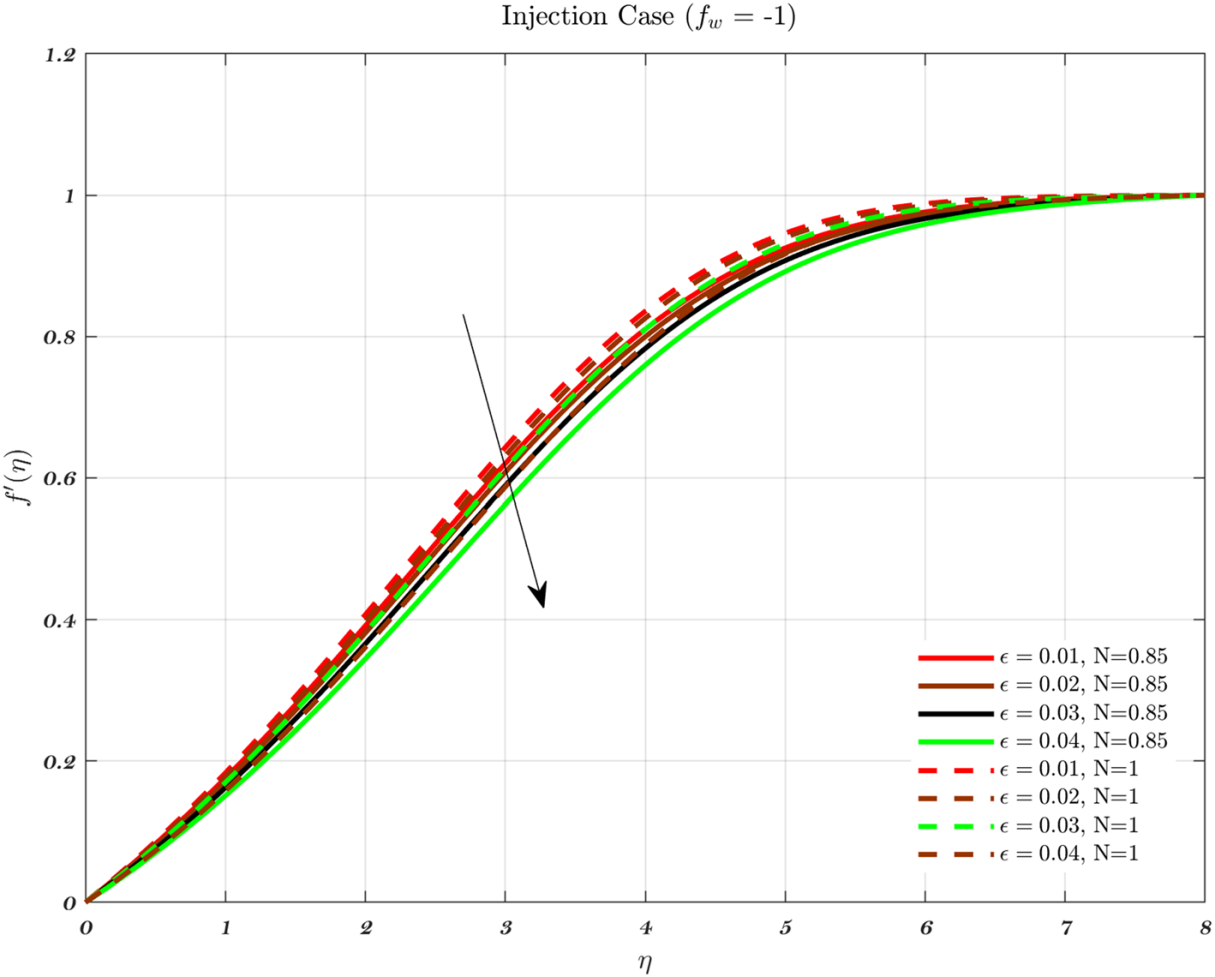
The influence of $\epsilon$ on the dimensionless velocity profile with the fixed parameters, $\varphi$, *Pr* and $a=0.01$ in case of Injection for both fluids.

**Figure 16:**
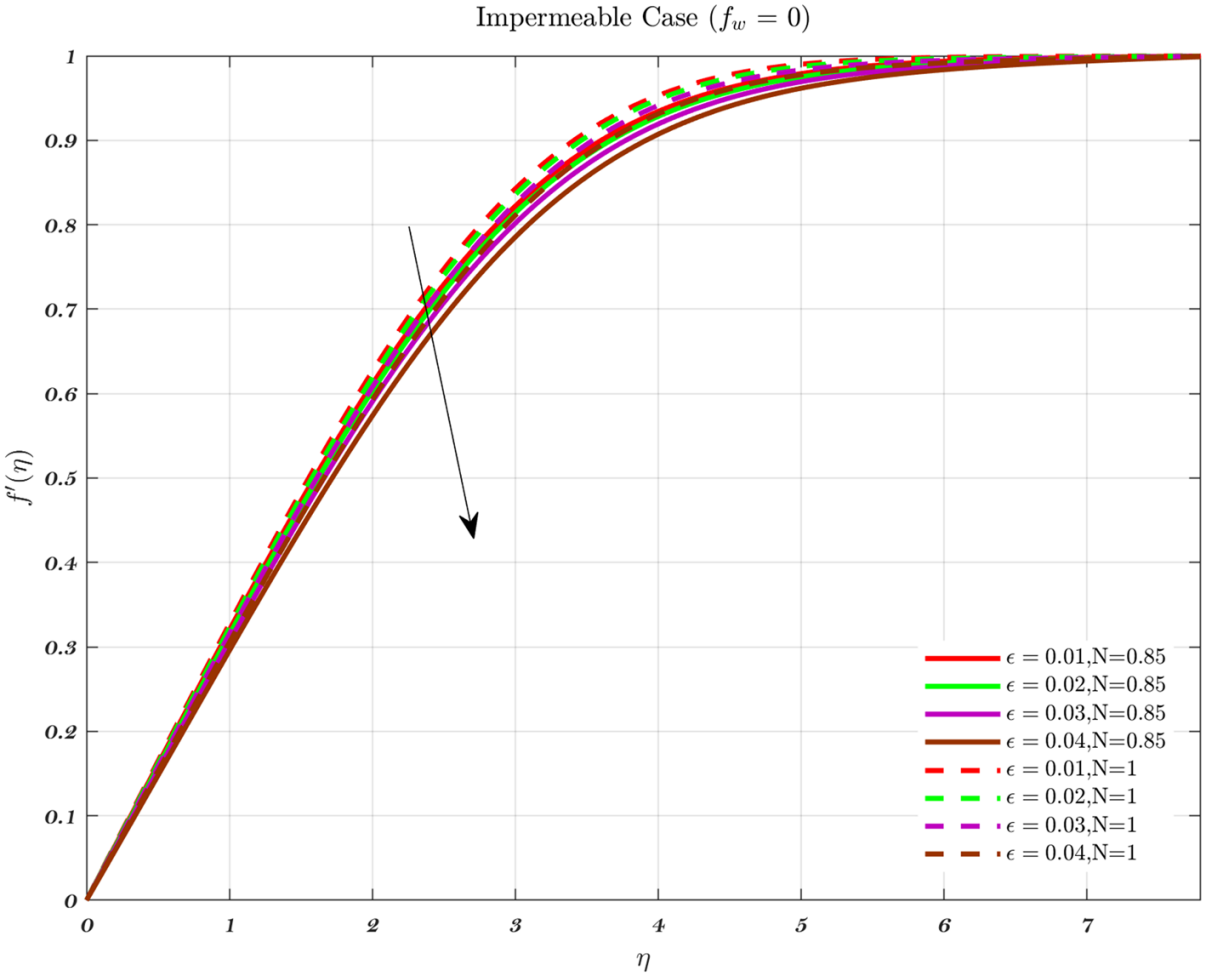
The impact of $\epsilon$ on the dimensionless velocity profile with fixed parameters $\varphi$, *Pr*, and $a=0.01$ for Impermeable surface for both fluids.

**Figure 17:**
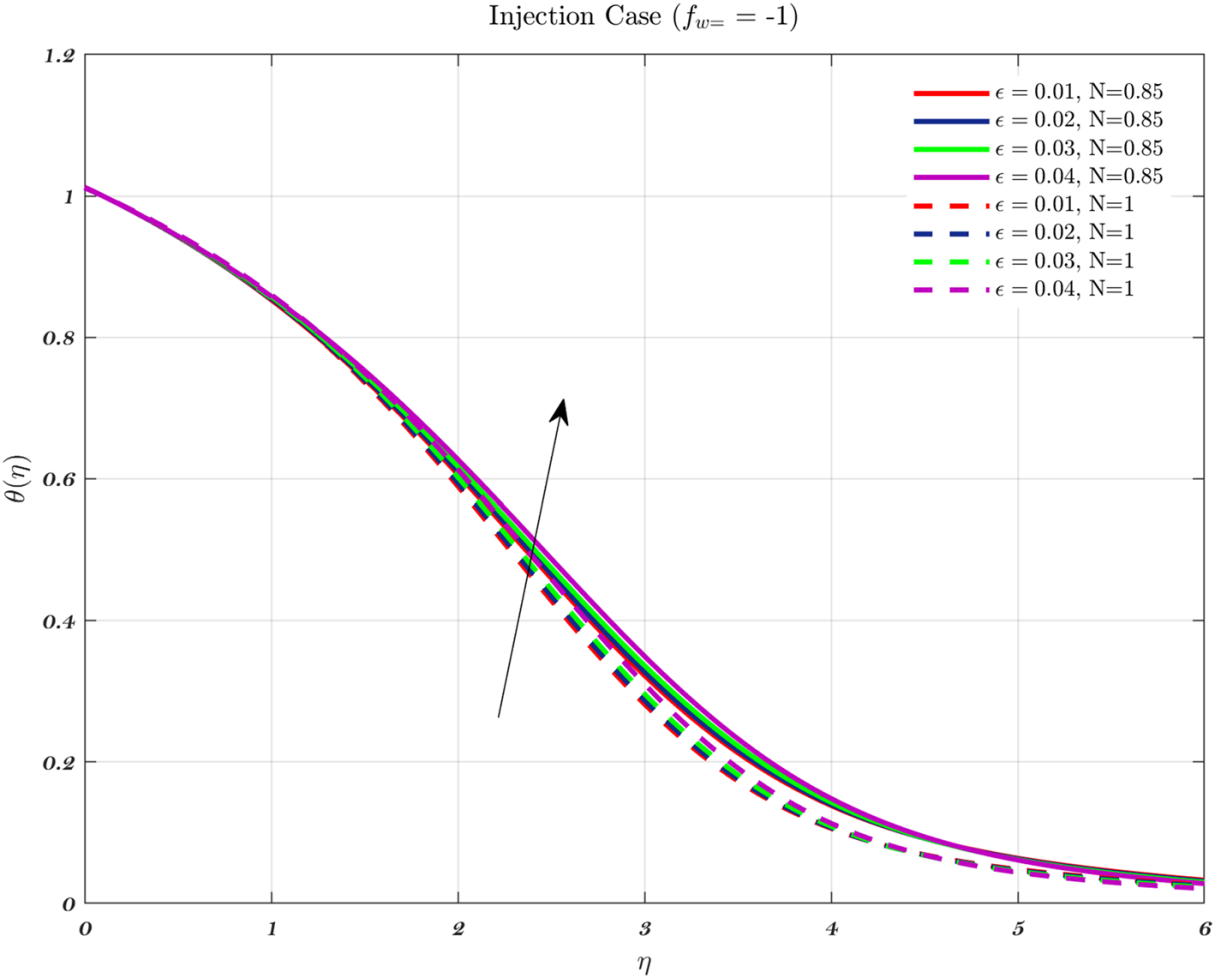
With $\varphi$, *Pr*, and $a=0.03$ held constant, examines the effect of $\epsilon$ on the dimensionless temperature profile for Injection using both fluids.

**Figure 18:**
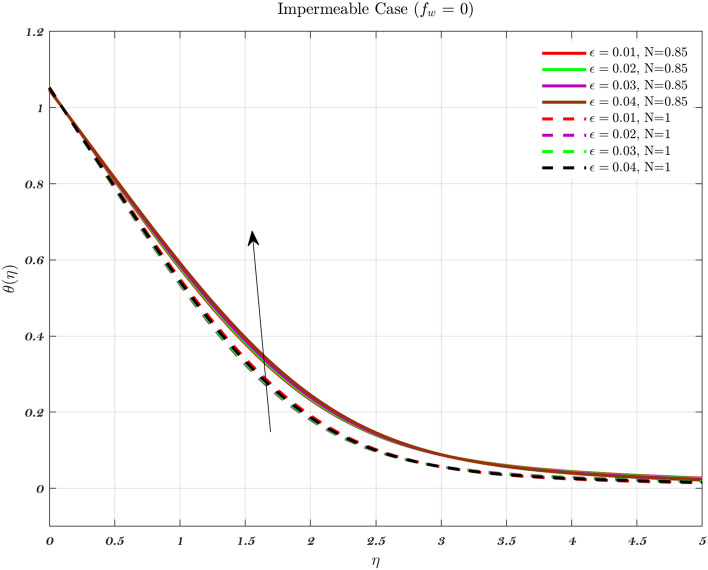
With $\varphi$, *Pr*, and $a=0.03$ held constant, examines the effect of $\epsilon$ on the dimensionless temperature profile for Impermeable surface using both fluids.

The velocity and temperature profiles for a number of different $\epsilon$ values are shown in Figs. 15, 16, 17 and 18 respectively. It has been observed that a boundary layer structure is formulated in the flow when the velocity $V_{W}$ is lower than the free stream velocity and $\epsilon$ is greater than 1. Therefore, physically speaking, the straining motion near the stagnation region increases, which in turn causes the acceleration of the external stream to increase, which leads to a decrease in the thickness of the boundary layer as $\epsilon$ increases^70^. When the stretching velocity of the surface is greater than the free stream velocity, an inverted boundary layer structure is created^70,71^ such BL creation does not occur when $\epsilon =1$. The stretching velocity is equivalent to the free stream velocity in the aforementioned phenomena^70,71^.

At the stagnation point, the kinetic energy term in the Bernoulli equation is nullified as a result of the absence of velocity. The dominance of static pressure and internal energy results in the fluid particle’s total energy. As the fluid departs from the stagnation point, it undergoes a redistribution of energy among kinetic energy, potential energy, and internal energy. This redistribution is influenced by the flow conditions and the presence of gravitational forces. These forces are particularly relevant in the context of the thin layer of fluid adjacent to a solid surface, where viscous effects play a significant role, see Figs. 17 and 18.

In the vicinity of the stagnation point, the boundary layer tends to exhibit a notably reduced thickness. The presence of a thin boundary layer has a direct impact on the velocity and pressure distributions surrounding the stagnation point. Consequently, these distributions have an influence on the energy equation. Understanding the redistribution of energy in a fluid as it moves along a surface requires a comprehensive analysis of the pressure and velocity gradients within the boundary layer.

The energy equation is a useful tool for analyzing the heat transfer process between a solid surface and a fluid medium. The rate of heat transfer and the development of the thermal boundary layer can be influenced by the temperature distribution within the boundary layer. This temperature distribution is dependent on the energy equation.

### Analysis with respect to convective parameter

**Figure 19:**
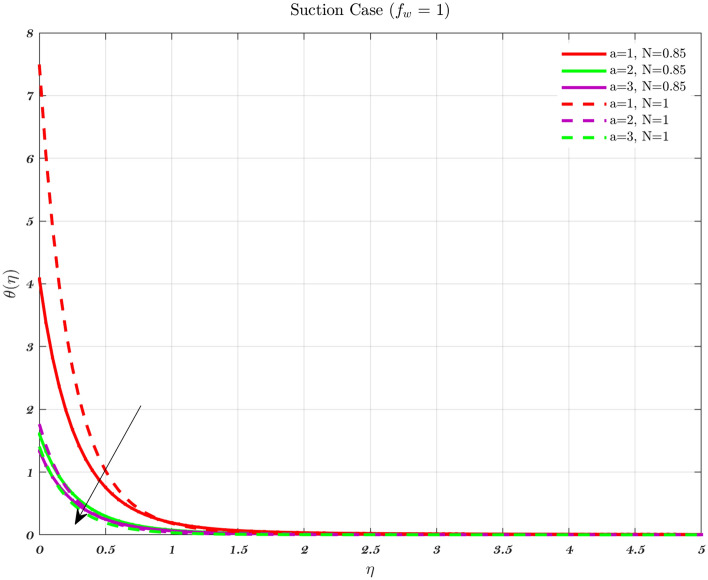
The influence of various values of the convective parameter *a* on the temperature profile in the case of Suction, for both flows.

**Figure 20:**
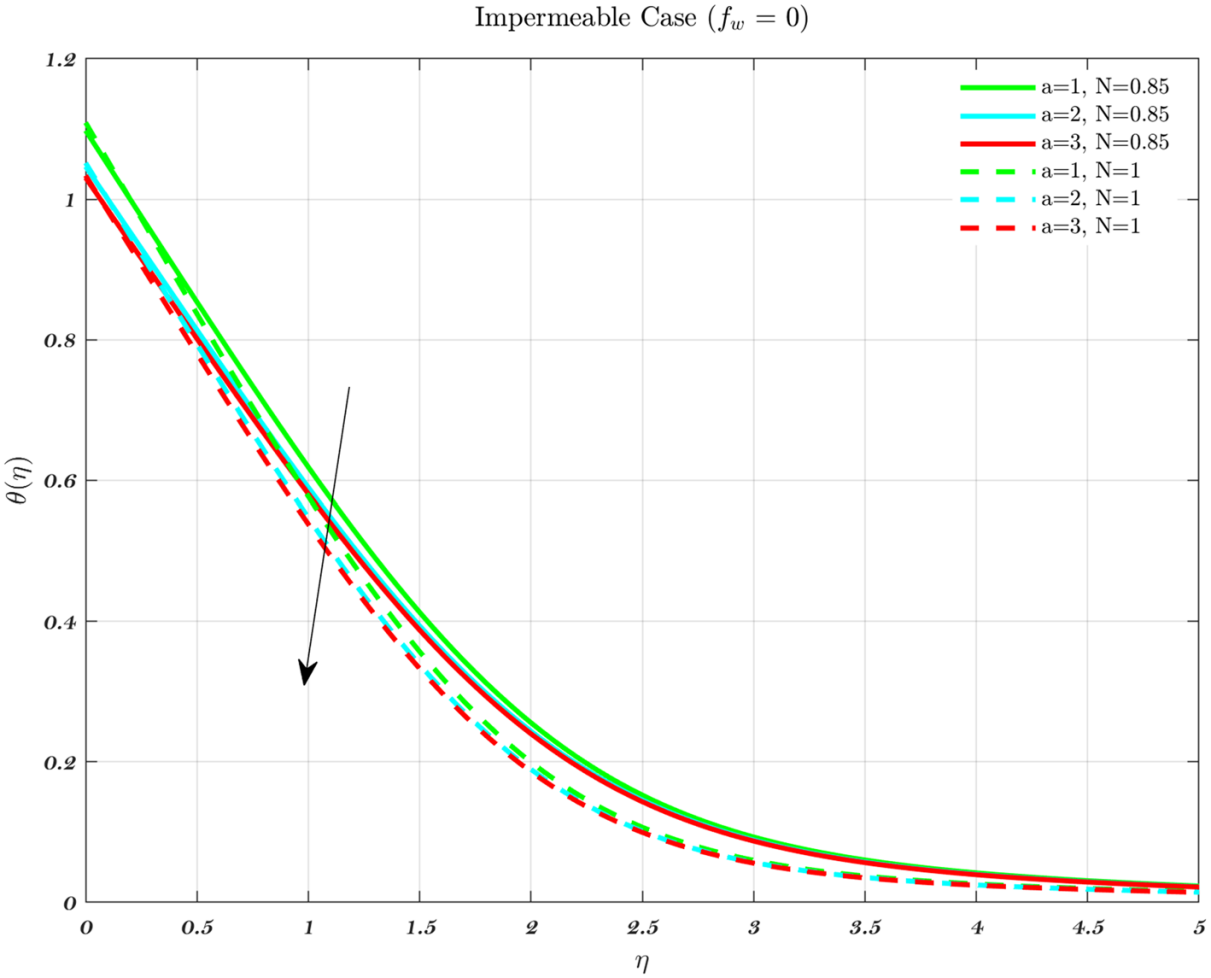
The influence of various values of the convective parameter *a* on the temperature profile in the case of Impermeable surface, for both flows.

The temperature profile for various values of a convective parameter *a* is provided in Figs. 19 and 20. It is observed that a higher value of *a* results in a lower non-dimensional temperature. These Figures depict results for the suction case to both Newtonian and non-Newtonian fluids, therefore the effect is applicable to all types of fluids. Increasing the value of the convective parameter results in an increase in the dimensionless surface temperature $\theta (0)$ therefore, the coefficient of heat transfer for the hot fluid which is denoted by $h_{f}$, has a relationship which is exactly proportional to the convective parameter at any given location^44,67–71^. An increase in the convective parameter leads to a decrease in the thermal resistance of the hot surface, which causes an increase in the dimensionless surface temperature. In addition, it is important to observe that as parameter *a* gets closer and closer to infinity, the $\theta (0)$ starts to creep closer and closer to 1. The injection procedure and the technique for an impermeable surface are identical.

### The effect of parameter modification on the Nusselt number and skin friction coefficient

**Figure 21:**
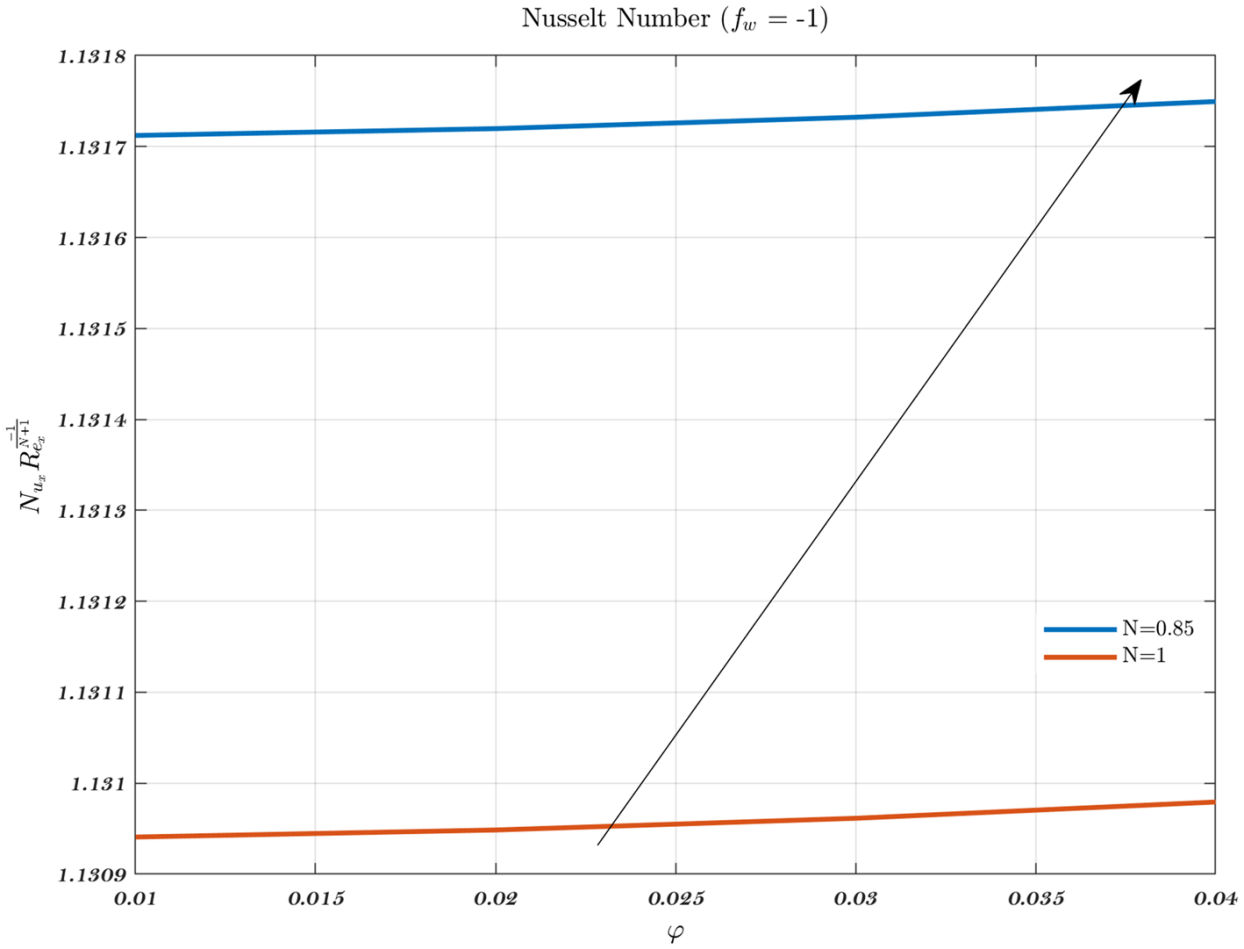
The influence of varying values of $\varphi$ on the Nusselt number in the context of an injection scenario involving two fluids.

The influence of various power-law fluids on the Nusselt number for impermeable surfaces, suction, and injection is seen in Fig. 21. It is observed that the presence of non-Newtonian fluid has an indisputable impact on the augmentation of the Nusselt number by considering the suction and impermeable surfaces. However, non-Newtonian fluid has a significant negative impact on heat transmission when used for injection. Newtonian and non-Newtonian fluids as well as their volume percentage do not have a significant influence on the local Nusselt number augmentation while either suction or injection is being performed. When compared to the other two scenarios, the Nusselt number and temperature gradient are both shown to be more favorable for heat transfer in the case of suction than they are in either of the other two scenarios.

**Figure 22:**
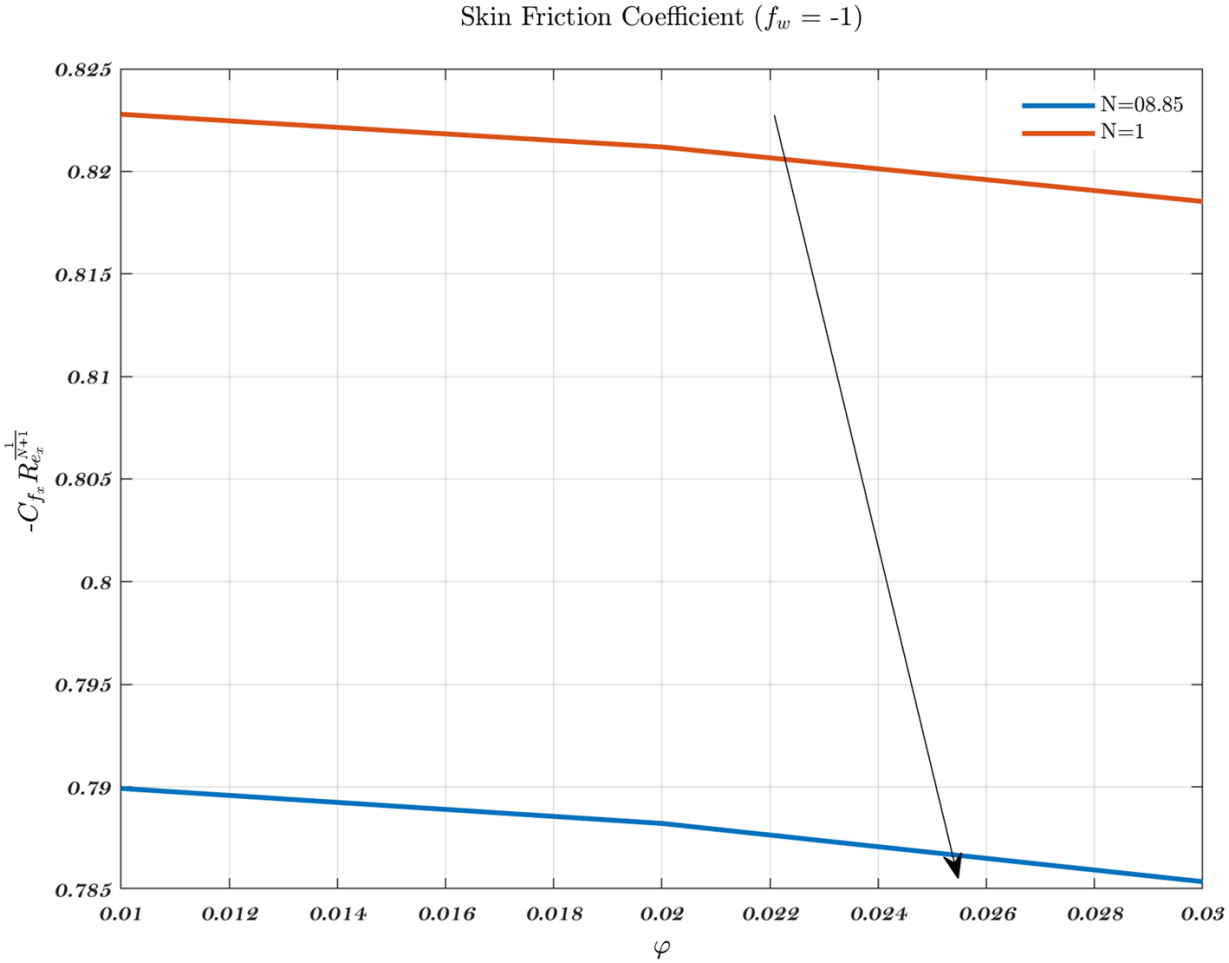
The influence of varying values of $\varphi$ on the Skin friction coefficient in the context of an injection scenario involving two fluids.

Figure 22 illustrates the effect on the local friction factor for two distinct classes of working fluids such as Newtonian and non-Newtonian. When compared to Newtonian fluid, the values of the local friction factor are found to be much greater than non-Newtonian fluid. Therefore, volume fraction augmentation is taken into consideration, the value of the local friction factor is highest for suction, while its value is lowest for injection.

## Conclusion

The governing equations for the partial differential equations related to synovial fluid have been effectively solved to provide insights into the convective diffusion of the viscous flow along the articular surfaces of the joints. This has been achieved by incorporating power-law fluids with varying permeability features and examining the stagnation point flow along a magnetic field. Because of its non-Newtonian nature, HA alters the reactivity of synovial fluid, although it makes up a small percentage of synovial fluid. Therefore, such presence may be estimated by considering both plasma and a reactant (which is essentially HA). A fluid whose characteristics alter when it interacts with a chemical agent whose mass fraction is considerably less than that of the fluid and whose diffusion velocity is much slower than that of the fluid, as a result, the diffusion is carried by the fluid at a speed close to that of the fluid itself. The findings are relevant not only to the flow of synovial fluid but also to the chemical interaction of HA with polymeric fluids. The flow of a shear-thinning, chemically-reacting fluid that may be utilized to represent the flow of synovial fluid is a major focus of study in this article. The most important results of this study are as follows:

- The boundary layer thickness is decreased by utilizing Newtonian fluid for suction and an impermeable surface, but the velocity gradient is unaffected. Non-Newtonian fluids have a greater velocity gradient across the surface during injection, and such difference is accentuated by increasing the fluid concentration^9,55^.
- The dimensionless temperature decreases initially for the suction case, which involves Newtonian to non-Newtonian fluids, and for the impermeable surface case, the temperature eventually rises.
- Physically, a rise in temperature of the synovial fluid because of the viscous nature of the joint tissues, which causes a portion of the input mechanical energy to be dissipated as heat.
- The synovial fluid’s composition and structure in which HA makes the fluid non-Newtonian, and its concentration affects synovial fluid responsiveness^48,59^.
- The rate of friction does not rely on the velocity of the SF. Though at high velocity or load, surface heating and viscoelastic processes might influence the tribology of the interface, the rate of motion does not directly impact the energy wasted per unit sliding distance under boundary lubrication conditions^71^.
- The use of a non-Newtonian fluid with a large volume fraction allowed for the decrease of the non-dimensional temperature on the impermeable surface. In the case of suction, the volume fraction does not have a significant impact on the surface temperature; however, using a non-Newtonian fluid (especially one with a low $n=N$) and increasing the suction parameter has a significant impact on both the surface temperature and the thickness decrease of the thermal boundary layer.
- In accordance with the principle of symmetry, the stagnation point for the flow field may be found precisely in the middle of the flow geometry. This body of research provides a reliable estimate of the strain rate at the stagnation point, supposing that the effects of flow modification are not very large^48,69,72^.
- It is important to observe that as parameter *a* gets closer and closer to infinity, the $\theta (0)$ starts to creep closer and closer to 1. The injection procedure and the technique for an impermeable surface are identical.
- An increase in temperature at the cartilage’s surface such that a heavier patient will have more knee contact during a given activity, creates more heat, which will lead to a rise in temperature, both within and around the knee joint.

## Data Availability

The data which support the findings of this study are available within the article.

## Abbreviations

x, y: Coordinate $\perp$ to sheet (m)
$u^{*}$, $v^{*}$: Velocity components along *x* & *y* directions (ms$^{-1}$)
$T^{*}$: Fluid’s local temperature (K)
$T_{\infty }$: Temperature far away from the sheet
$\rho _{bf}$: Effective base-fluid density (kg m$^{-3}$)
$\rho _{f}$: Pure fluid density (kg m$^{-3}$)
$\mu _{bf}$: Base fluid’s effective dynamic viscosity (kg m$^{-1}$ s$^{-1}$)
$k_{bf}$: Base fluid’s thermal conductivity (W m$^{-1}$ K$^{-1}$)
$$P_{r}$$: Prandtl number
$$\varphi$$: Volume fraction
*a*: Slip parameter
*K*: Permeability
$$\alpha$$: Dimensionless parameter
$U_{\infty } \dfrac{d U_{\infty }}{dx}$: Stagnation point flow term
$C_{f}$: Skin Friction coefficient
$Re_{x}^{1/2} C_{f}$: Reduced Skin Friction coefficient
$Re_{x}^{-1/2} Nu_{x}$: Reduced Nusselt number
